# Permanent Supportive Housing Design Characteristics Associated with the Mental Health of Formerly Homeless Adults in the U.S. and Canada: An Integrative Review

**DOI:** 10.3390/ijerph18189588

**Published:** 2021-09-12

**Authors:** Kimberly A. Rollings, Christina S. Bollo

**Affiliations:** 1 Department of Psychology, School of Architecture, University of Notre Dame, Notre Dame, IN 46556, USA; 2 Health and Design Fellowship Program, Institute for Healthcare Policy and Innovation, University of Michigan, Ann Arbor, MI 48109, USA; 3 School of Architecture, University of Illinois Urbana-Champaign, Urbana-Champaign, IL 61820, USA; cbollo@illinois.edu

**Keywords:** homelessness, permanent supportive housing, Housing First, Treatment First, mental health, behavioral health, built environment, architecture, design, integrative review

## Abstract

The built environment directly and indirectly affects mental health, especially for people transitioning from long-term homelessness to permanent supportive housing (PSH) who often experience co-occurring behavioral health challenges. Despite a rapid increase in PSH availability, little research examines influences of architecture and design within this context. This integrative review synthesized limited research on PSH design in the U.S. and Canada to identify built environment characteristics associated with PSH residents’ mental health, highlight gaps in the literature, and prioritize future research directions. A systematic search for peer-reviewed articles was conducted using nine databases drawing from multiple disciplines including architecture, environmental psychology, interior design, psychology, psychiatry, medicine, and nursing. Seventeen articles met inclusion criteria. Study design, methodology, built environment properties, place attributes, and relevant findings were extracted and iteratively analyzed. Three domains relevant to architecture and design were identified related to home, ontological security, and trauma sensitivity; dwelling unit type, privacy, control, safety, housing quality and location, and access to amenities; and shared common space. Integrative review results emphasize the potential of architecture and design to contribute to improved built environment quality and mental health outcomes among PSH residents. Methodological limitations and directions for future research are also discussed.

## 1. Introduction

The built environment directly and indirectly affects mental health, especially for permanent supportive housing (PSH) residents transitioning from long-term homelessness who often experience co-occurring behavioral health challenges. PSH aims to engage and rapidly house individuals experiencing chronic homelessness while providing flexible and voluntary services needed to improve health, housing tenure, and financial stability. Despite the recent increase in the construction and renovation of PSH facilities in the United States and Canada, little research examines influences of architecture, design, and the built environment within this context. Understanding how these influences support or hinder PSH program goals and resident mental health is especially urgent considering increasing rates of homelessness, mental disorders, and mental illness; an aging and growing population; and the high construction or renovation costs of establishing a new PSH building [1]. The purpose of this integrative review was to synthesize limited research on PSH design and identify built environment characteristics associated with PSH residents’ mental health; highlight gaps in the literature; and prioritize future research directions. The following background section provides context about long-term homelessness in the U.S. and Canada, co-occurrence of homelessness and mental illness, PSH and service models, and existing mental health and built environment literature. Remaining sections describe the literature search and analysis process, results and interpretation, and directions for future research. 

## 2. Background 

### 2.1. Chronic Homelessness in the U.S. and Canada

Homelessness is a prevalent social and public health concern in the U.S. and Canada, as well as many other developed countries [2]. Of the estimated 580,466 people in the U.S. who experience homelessness on a single night, more than one-quarter experience chronic homelessness for a period greater than 12 months or endure at least four episodes of homelessness totaling 12 months over the previous three years [3]. U.S. rates of chronic homelessness rose 15 percent between 2019 and 2020 and, as of 2020, 66% of people experiencing chronic patterns of homelessness were counted living in unsheltered locations [3]. Similarly, more than 235,000 people experience homelessness annually in Canada, and an estimated 25,000–35,000 people experience homelessness nightly [4]. Four to eight thousand people experience long-term homelessness over the course of a year and six to 22 thousand experience repeated episodes of homelessness at some point during a given year [4]. Individuals experiencing chronic homelessness are more likely to live unsheltered on the street or in a location not meant for human habitation such as an automobile, abandoned building, or park [3]. These individuals are at higher risk for exposure to violence, victimization, drug use, and the elements (e.g., sun and heat, freezing temperatures, rain); lack bathing and toileting facilities, adequate sleeping accommodations, refrigeration for food and medicine, cooking facilities, privacy, and a location for hosting guests and social interactions; and do not have a stable address needed to receive services and mail [1]. These challenges, along with co-occurring behavioral health issues and a lack of a safe, stable environment for recovery, often result in recurrent loss of housing [5] and perpetuate the cycle of homelessness.

### 2.2. Homelessness and Mental Health

Adverse mental health refers to negative affect and emotions, psychological distress, and psychiatric disorder and illness [6]. Homelessness and mental health share a bi-directional relation such that homelessness may contribute to or exacerbate poor mental health and mental illness, and consequences of mental illness may lead to homelessness [7]. Disorders that affect people experiencing or transitioning from long-term homelessness often include schizophrenia, anxiety, and depressive and stress-related disorders [8]. The combination of homelessness and mental illness increases the risk of substance use and abuse [9]. In the U.S., an estimated 25–30% of people experiencing chronic homelessness also have a serious mental illness, 45% have any mental illness, and 35% have chronic substance use issues [10,11,12,13]. By comparison, an estimated 4.2% of the general U.S. adult population is diagnosed with a serious mental illness [13]. In Canada, estimates indicate that 30–35% of those experiencing homelessness, and up to 75% of women experiencing homelessness, also have a mental illness [4]. People experiencing chronic homelessness often face higher incidences of these co-occurring behavioral health challenges, as well as repeated instances of early-childhood, adolescent, or adult trauma; physical, psychological, or emotional abuse; domestic violence; poverty, disability, and significant degrees of social isolation; and have little or no access to medical, dental, and mental health services [1,14,15]. These complications often lead to a need for high levels of supportive services, emergency shelter space, and emergency services [4]. As individuals transition from homelessness to permanent housing, the design of the physical setting as well as supportive services must address these complex needs. Housing provides a stable platform from which other physical, mental, and social issues can begin to be addressed. 

### 2.3. Permanent Supportive Housing (PSH) and Service Models 

The U.S. and Canada frequently address chronic homelessness via PSH [2,16,17,18]. PSH provides people experiencing chronic homelessness, disability, and behavioral health issues with immediate access to affordable housing and integrated, continuous, and comprehensive support services without limits regarding length of stay [1,19]. PSH dwelling units are intended for long-term tenancy while overnight shelter and transitional housing typically limit stays to one and 90 days, respectively. Participation in on-site PSH supportive services (e.g., case management, health care access, substance use treatment, mental health counseling, support groups, life skills training, social programming, and employment search assistance) is encouraged. Although the evidence supporting PSH effectiveness for single adults and families is mixed [1,19,20], residents typically report increased levels of freedom, autonomy, choice, and control, and a majority of clients participate in on-site services [21,22]. An extensive literature discusses key PSH elements and effectiveness [1,19], as well as the development of and differences between “supported” and “supportive” housing [23].

PSH employs one of two service models: Housing First or Treatment First. Housing First (HF) offers immediate housing and supportive services to individuals experiencing homelessness and behavioral health issues with few or no preconditions; individuals are encouraged to define their own recovery-oriented goals, including if, when, and how they access supportive services [1,24]. Conversely, Treatment First (TF) offers housing to the same population only after successful completion of required treatment, and with sobriety and abstinence as preconditions. TF residents are expected to move along a continuum of housing options—from the street to overnight shelters, from shelters to transitional housing, and from transitional to permanent and autonomous housing—as their recovery progresses [25]. Additional information about Housing First and Treatment First approaches, as well as their history, is available elsewhere (e.g., [2,17]).

Regardless of service model, funding for PSH programs and facilities includes a complex combination of federal, state, and other public and private sources. These funding sources have increased the number of PSH beds available in the U.S., doubling from 188,636 to 373,030 between 2007 and 2020 [3]. Despite this widespread increase in PSH and Housing First in the U.S. and Canada, an analysis of the architectural design and built environment is lacking [26,27]. A large body of literature assesses the PSH model, but rarely the space and place in which those programs occur. The high cost of land and construction of a new PSH building or the acquisition and renovation of an existing building [1] require understanding how the physical environment of these facilities—their architecture, design, and built and natural elements—can support or hinder tenant recovery goals, mental health outcomes, and PSH program success.

### 2.4. Architecture, Design, the Built Environment, and Mental Health

A growing body of literature documents direct and indirect effects of architecture, design, and the built environment on mental health (see reviews [6,8,28,29,30,31,32,33]). Direct mental health effects result from, for example, indoor and outdoor toxins and air pollutants, daylight, noise levels, air quality, and indoor temperature; indirect effects of built environment characteristics influence mental health via effects on stress, recovery from cognitive fatigue and stress, and control of psychosocial processes including control, identity, insecurity, and social interaction and formation of supportive relationships [34,35,36]. Research suggests that design characteristics such as housing quality, building location, furniture arrangement, building floor plan layout, and access to nature and outdoor space contribute to these indirect effects. Although this literature largely excludes people transitioning from homelessness, results offer promising directions for future PSH built environment research and design. Housing quality influences physical and mental health among populations who have and have not experienced homelessness and co-occurring behavioral health challenges [6,34,37,38]. Within the supported housing literature, both objective and perceived poor housing quality are associated with maladaptive behaviors, reduced quality of life, and decreased global functioning [35]. Concerns about poor housing quality, including cold temperatures, damp or poor air quality, and infestation, indirectly affect mental health by increasing stress and depressive symptoms [6,39,40,41]. Furthermore, poor housing quality and maintenance can contribute to stigma and negative personal identity as one’s home is a reflection of one’s self [6]. By extension, these findings are particularly relevant to PSH residents working to reconstruct their identities while simultaneously at higher risk of living in poor quality housing. The social context and physical location of housing also indirectly affects mental health and well-being via availability of and proximity to public transportation and community services, perceived and actual safety, and neighborhood physical condition [6,35,36,42].

Within residential spaces, prior research demonstrates that furniture arrangements and floor plan layouts indirectly affect mental health by influencing social interaction, isolation, privacy, and control. Circular or “socio-petal” furniture arrangements encourage use and social interaction when compared to linear or “socio-fugal” arrangements [43]. The arrangement of spaces within a home or building can also influence occupants’ ability to control social interaction [6,44]. Smaller clusters of dormitory rooms that share common space, when compared to long hallways known as “double-loaded corridors” with rooms on either side, better promote social interaction, support, and cooperation [45,46,47]. These building layouts offer transitional semi-public and semi-private spaces that allow occupants to avoid crowding, isolation, and social withdrawal, and facilitate control of desired levels of privacy and interaction [6,35,45,48]. Interaction “nodes” (e.g., mailboxes, shared laundry facilities) and high-use pathways within floor plans also affect interaction patterns [49]. Similarly, shared common areas (when designed appropriately for size, location, visibility, and décor) play a role in providing this spatial hierarchy and choice, as well as creating community and decreasing isolation [6,27,45]. Additionally, access to nature and outdoor space affect mental health and interaction in residential settings [35,50]. In summary, housing quality, proximity to transportation and necessities that support independence, furniture arrangements and floor plan layouts that promote interaction but allow for privacy and access to nature and outdoor space that promotes both recovery from stress and social interaction are especially relevant to PSH residents working to regain control of their lives and rebuild socially supportive relationships.

Considering the co-occurrence of mental illness and homelessness among PSH residents, surprisingly little research focuses on how PSH resident mental health is affected by the PSH built environment. With the exception of some prior work on shelter and transitional housing settings [51,52,53], much of the built environment and adverse mental health literature examines clinical and institutional behavioral health settings [54,55,56] or supported housing for people with mental illness and disabilities who have not also experienced homelessness [30,35,57,58,59]. Therefore, the following review of the limited research on the architecture, design, and built environment of PSH aimed to identify built environment characteristics associated with PSH residents’ mental health, recognize gaps in the literature, and prioritize future research directions.

## 3. Methods

### 3.1. Review Aims and Design

This integrated review synthesized literature addressing design aspects of the PSH built environment associated with mental health outcomes of adult residents formerly experiencing homelessness in the U.S. and Canada. An integrative review approach was chosen because the process allows for analysis of findings from a diverse range of research methodologies to capture a more comprehensive understanding of the phenomenon of interest [60]. Whittemore and Knafl’s [60] five-part integrative review methodology was followed: (1) problem identification, (2) literature search, (3) data evaluation, (4) data analysis, and (5) result presentation. Specifically, the data analysis step systematically followed data removal, data display, data comparison, conclusion drawing, and verification processes suggested for integrated results [60,61]. The research question identified for this literature review was: What architectural design and built environment characteristics contribute to mental health outcomes among adults formerly experiencing homelessness living in permanent supportive housing?

### 3.2. Literature Search Strategy and Inclusion Criteria

Prior to conducting the literature search, selection criteria were prepared. Inclusion criteria were: (1) peer-reviewed papers published in domestic or foreign academic journals and in the English language from 1970 onwards; (2) quantitative, qualitative, and mixed-methods studies or literature reviews; (3) papers addressing architecture and the built environment, mental health, long-term homelessness, and single-site supportive housing in the U.S. and Canada; (4) studies sited in facilities for individuals at least 18 years of age living independently, as facilities that provide housing for families and children vary in unit types, architectural program, and needed amenities and services; and (5) single-site, rather than scattered-site, supportive housing due to the rapid construction, renovation, and implementation of this housing type in the U.S. and Canada [13]. When compared to scattered site, single-site facilities more often require architectural and design services [62]. The U.S. and Canada were selected due to similarities in PSH program and facility expansion, as well as policies and available resources related to homelessness and mental health. For example, the two countries were early adopters of Housing First policies [63]. Approaches to addressing homelessness and mental health, including care and service delivery and models based on “housing as a human right,” in other industrially established countries and regions (e.g., Europe, Scandinavia, Australasia, and Japan) differ from North America and were beyond the scope of this review [2,64,65].

The exclusion criteria for articles in the literature review were: (1) papers other than peer-reviewed original research and review papers (conference papers and proceedings, dissertations and theses, grey literature); (2) articles focused on inpatient settings (e.g., psychiatric wards), clinical and institutional treatment settings, and nursing homes and eldercare environments; (3) papers focused on group or halfway housing, temporary shelter, and emergency or post-disaster housing, including for refugees and survivors of domestic violence; and (4) studies focused on populations experiencing intellectual or developmental disability.

Two independent researchers conducted the search between 23 March 2021 and 6 May 2021. Customized search syntax was created for nine databases in nursing (CINAHL-Cumulative Index of Nursing and Allied Health Literature; Health Source), architecture and interior design (JSTOR), psychology (PsycInfo, Social Service Abstracts), and medicine (PubMed), and covering multiple disciplines (Scopus, Web of Science). Keywords focused on three concept domains: supportive housing, mental health, and design. Relevant subject headings were used to inform keyword selection, and titles and keywords of relevant papers were reviewed to identify frequently used terms. Resulting search syntax consisted of the keywords illustrated in Table 1 (complete syntax in Table S1, Supplementary Materials). Additional database-specific filters were applied as available and relevant to inclusion and exclusion criteria (e.g., discipline, subject, publication type, peer-review status, and population age).

Literature retrieved from each database was catalogued using bibliographic management software (EndNote 20), reviewed, and organized after combining records and removing duplicates. Each researcher independently identified, reviewed, and selected relevant literature for eligibility assessment following PRISMA’s four-step guidelines for systematic reviews [66]: identification, screening, eligibility, and inclusion. Titles and abstracts of literature identified via database searches were screened according to the previously stated inclusion and exclusion criteria. Researchers’ screened articles were compared for duplication and agreement. The full text of each relevant screened article was then assessed for eligibility in detail by both researchers. Reference lists of eligible studies were also mined to identify additional relevant articles and were subjected to screening and eligibility assessment. Final articles were selected for inclusion in the review after researchers reached a consensus concerning any disagreements in eligibility assessment.

**Figure 1:**
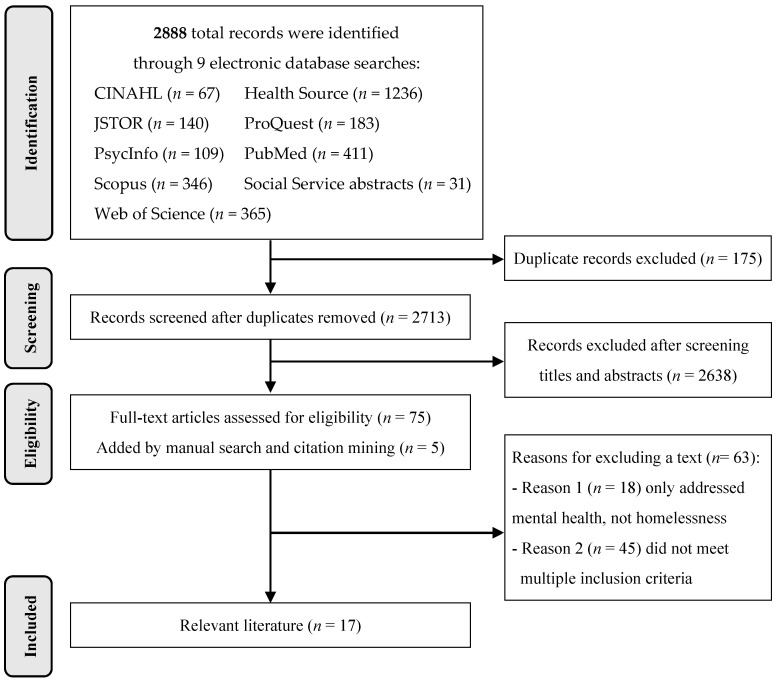
PRISMA flow diagram [66] illustrating the database search and article selection process.

Figure 1 illustrates the identification, screening, eligibility assessment, and inclusion process. From the 2888 papers identified by the researchers, 175 duplicates were removed. Another 2638 documents were excluded after screening titles and abstracts. For eligibility assessment, two co-researchers recorded whether the full-text met inclusion criteria via a matrix [67]. Discrepancies were discussed until a consensus was reached: of the remaining 75 documents, 63 were deemed ineligible because they focused only on mental health and not homelessness (18) or did not meet multiple inclusion criteria, such as location or context (45). In addition to the remaining 12 eligible articles, five articles were identified via mining reference lists for eligible articles, resulting in a total of 17 papers included in the review.

### 3.3. Literature Evaluation and Analysis

After selecting literature for inclusion, a second, more comprehensive, matrix was used to extract and compile predetermined and relevant data [60] on the following categories: article type (study or review), study type and design (quantitative, qualitative, mixed methods, review), data collection methods, participant sampling and characteristics, study results relating to the built environment, and limitations, as well as housing type, service model (Housing First or Treatment First), site context (single or scattered site), and dwelling unit types (independent or shared independent apartments, congregate housing, SRO). Data were compiled according to the definitions presented in Table A1 (Appendix A). Articles were then assigned relevant spatial scales (defined in Appendix A) based on the built environment characteristics addressed: room (dwelling or shared common areas), building, and location.

Built environment properties (Table A1) and place attributes Table A2 (Appendix A) were also extracted from independent and dependent variables and study results. Built environment properties and attributes ranged from direct measures (e.g., housing quality) to mentions in participant responses (e.g., desire for more privacy). Built environment properties addressed within each article were extracted and then classified as physical, ambient, or spatial (defined in Appendix A). Socio-spatial “place attributes” were extracted and coded according to definitions in Table A2 (Appendix A). These attributes, largely from the environmental psychology literature, offer a useful perspective and lexicon for coding dynamic experiential interactions, transactions, and relations between the social and physical environment [68,69]. In other words, these attributes have both social and physical environment components that contribute to occupant experience. The environmental psychology perspective aids in understanding how social and physical environments shape experiences and outcomes, including health, yet the experience of homelessness is not often explored using this perspective [70].

Research design and methodological variations in articles necessitated coding for two additional criteria relevant to this review: built environment relevance and methodological rigor. Articles were assigned to one of four “relevance” categories according to the level at which the built environment was a focus in the research design. Four measures of methodological rigor were created due to variations in article types (study, review), study design, and methodology. Table 2 define each built environment relevance category and methodological rigor rating level. Relevance categorizations and rigor ratings were assigned after completion of coding and informed the analysis stage.

Two researchers independently evaluated each included paper, extracted data and entered details into matrices, and iteratively coded content, cross-checking within and between articles and researchers and discussing discrepancies until consensus was reached. The matrix containing all extracted data organized by category was used by each researcher to iteratively code each article for built environment properties, place attributes, and built environment study results. As data were conceptualized at higher levels of abstraction, articles were re-reviewed to confirm coding accuracy and completion within the revised conceptualizations. Analysis consisted of examining study results, built environment properties, place attributes, and rigor. Common built environment patterns were identified and discussed before grouping into overall domains for presentation. Analyses results and domains were discussed until shared conclusions were reached.

## 4. Results

### 4.1. Overview of the Included Literature

Table 3 summarizes study location, purpose, research design, rigor, participants, service model, site, dwelling type, and spatial scales of the 17 reviewed articles. One review and 12 studies were based in the U.S. and 4 studies were located in Canada. Publication dates ranged from 2005 to 2021, which coincides with the inception and increase in PSH in the late 1990s and early 2000s. Nine of the included papers were published since 2016, indicating that work on PSH design and the built environment is largely recent and exploratory.

#### 4.1.1. Research Design, Participants, and Rigor

The reviewed literature contained studies of varying research design that used quantitative, mixed, and qualitative methods, in addition to one non-systematic review paper (Table 3). Four quantitative studies followed quasi-experimental, repeated measures, or comparative designs. Data collection included questionnaires, systematic observer ratings, publicly available data, and structured interviews. Three papers used mixed methods, implementing case study, comparative, and explanatory sequential designs with similar quantitative data collection methods, as well as open-ended interviews, space syntax, and photography. The nine remaining papers used qualitative methods including case study, exploratory, and grounded theory design. Thematic and primary cycle analyses were used to analyze ethnographic shadowing, interview (semi-structured, open-ended, life-history, and in-depth), focus group, and participant drawing data. Thirteen studies used cross-sectional and three used longitudinal (2–4 years) designs.

Study participant distribution ranged from 103 to 756 (4 quantitative studies), 24 to 38 (3 mixed-methods studies), and 10 to 39 people with one outlier at 106 people (9 qualitative studies). Sampling was largely convenience (10 including 1 provider-recruited sample) and purposeful (3 purposeful, 2 criterion), with only one random sample. Although participant ages ranged from 18 to 70 years across all papers, the average age of study participants across 12 of 16 studies was younger (42–56 years), highlighting a need for work with older adults. One additional article focused on young adults (23 years of age, on average), two studied older adults (60 years of age, on average and 55–70 years), and two papers did not specify participant age. Participant gender also tended to be more than half male (11 studies), although two were majority female, two focused on only male or only female participants, and one was not specified. Participant racial and ethnic information was specified by 11 articles: three participant samples were less than 40% white (with one at 0%) and eight were at least 40% white. At initial data collection, participants were housed between two months and four years, on average, ranging from just before being housed to 23 years among the 11 studies that reported the amount of time participants had been housed.

Following the integrative nature of the review, rigor ratings were completed separately for the review paper and quantitative, qualitative, and mixed-methods studies. Half of both quantitative (2 of 4) and qualitative (4 of 9) studies were of high rigor, mixed-methods studies were of low (2) and medium (1) rigor, and the single review article was of low rigor. Other than the housing quality measures noted in Section 4.3.4, no standardized, validated instruments were used to evaluate the PSH built environment. Moreover, cross-sectional studies and convenience samples lacked randomization and precluded generalizability and causal conclusions among quantitative and mixed-method studies. Nonetheless, the limited literature suggested built environment factors and insights for future research, especially qualitative studies of high rigor.

#### 4.1.2. Housing Type, Service Model, and Spatial Scale

Housing information (service model, site, and dwelling unit) was documented (Table 3) to understand the range of facilities and models being compared to single-site PSH. Ten studies focused solely on single-site PSH, while five papers included both single- and scattered-site projects. Two studies did not explicitly articulate site context but presented enough information to assume inclusion of single-site supportive housing. Eleven studies specified that a Housing First model was implemented, and three of those studies compared Housing First to Treatment First (TF) approaches.

The “Scale” column in Table 3 notes the spatial scales addressed by each paper. The most commonly addressed design characteristics were at the room (13 dwelling, 7 shared spaces) and building (10) scales. Although this review’s aim focused on room and building scales as they are most influenced by architectural design, more than half (11) of the included articles also addressed location. This prevalence suggested that PSH location characteristics are worthy of consideration when evaluating and designing PSH built environments. Eight studies addressed built environment characteristics at all three spatial scales, but only one paper addressed all three scales including both dwelling unit and shared space [71]. The presence of all three scales across the limited number of studies indicated that PSH research and design need to address multiple scales and interactions between scales on relevant project outcomes.

#### 4.1.3. Built Environment (BE) Relevance

Table 3 presents the identified literature organized by built environment relevance category. Although new and renovated PSH facilities are being rapidly deployed, only two reviewed papers were “design driven” and aimed to inform architecture and design [27,71]. The design-driven papers focused on shared common space design (location, visibility), use, and role in recovery via mixed methods [27]. The review paper addressed the interaction between program operations (house rules, mobilization of peer support, role of professional services, and cultivation of a shared approach to recovery) and setting (appearance, location, design for sociability and personal space, and facility oversight, security, and upkeep) on program outcomes (e.g., sobriety), as well as offered informal design recommendations [71].

The eight “built environment (BE)-focused” papers assessed built environment factors without aiming to assess or inform design, which is necessary for the translation of research to practice. BE-focused papers included quantitative, mixed, and qualitative studies. Quantitative built environment studies focused on housing quality, preferences, satisfaction, and associated outcomes (Section 4.3.4). Two mixed-methods BE-focused studies examined housing and neighborhood quality and satisfaction [72], and perceived and objective measures of neighborhood safety [73]. Qualitative BE-focused studies examined common space use, structure, and experience [26], and macro- (housing policy, trauma-sensitive env), meso- (social relations), and micro- (behavior coping strategies) level influences on women’s mental health (stress-related sleeping issues, mental health symptoms, isolation from fears of violence and the drug-sex economy), drug use, and housing stability [36].

Six qualitative studies were categorized as “inductive” and addressed the built environment via emergent themes from participant responses. These studies explored experiences of housing type, neighborhood, and housing stability [77]; how physical and social environments contributed to feelings of home and social exclusion among older men formerly experiencing homelessness [78]; what made a house feel like a home with respect to identity reconstruction, housing stability, and community engagement [79]; the physical, social, economic, and policy environment, housing environment perception, and substance abuse risk [80]; ontological security markers and mental health social relationships, and positive identity [81]; and markers of ontological security based on treatment type and life history [82]. One additional qualitative study was classified in a fourth relevance category, “mentions.” The study qualitatively explored associations between events, residents, staff, and neighbor interactions, and community interaction. Participant responses contained relevant built environment factors aligned with review aims [83].

#### 4.1.4. Built Environment Findings, Properties, Place Attributes, and Integrative Domains

**Figure 2:**
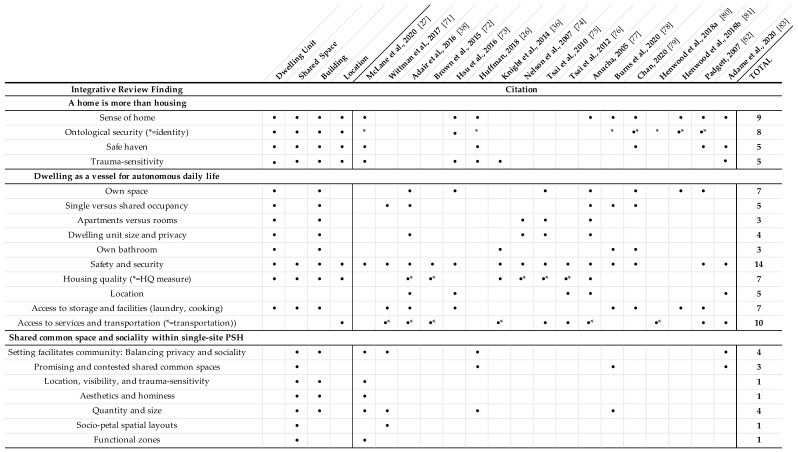
Integrative review findings by article and spatial scale implications.

The Supplementary Materials contain a summary of built environment results (Table S2a,b), physical and ambient properties (Table S3), and place attributes (Table S4) extracted from all 17 papers. Extracted built environment properties and place attributes varied in scope from those that were central to the research design (e.g., shared common space [27]; safety and home [73,79]) to aggregated housing quality measures to participant discussion at various levels of detail via interview and survey responses. Because the level of and methodological approach to exploration of these built environment properties and place attributes varied greatly, and were often not included in article study designs or discussed in detail with study participants, the existing literature did not support integrative conclusions regarding properties and attributes beyond those articulated in Figure 2. In aggregate, however, the appearance of these built environment properties and place attributes (and absence of others) across articles suggested directions for future research to build upon the limited literature and inform PSH design. Detailed matrices and coding results informed identification of three domains of integrative review findings outlined in the subsequent sections. Figure 2 summarizes analyses results organized by three domains, each with implications for architecture and design: A home is more than housing; dwelling as a vessel for autonomous daily life; and shared space and sociality within single-site PSH. Within these domains are built environment factors across the three spatial scales of room (dwelling and shared common space), building, and location.

### 4.2. A Home Is More Than Housing

Analyses of extracted built environment data indicated that effects of the PSH built environment on mental health resulted not only from the benefits of having permanent shelter, but also creating a home; having one’s own quality place to recover, redefine identity, and acquire skills; and building community by forming socially supportive relationships. The first domain, “a home is more than housing” (Figure 2), captured built environment properties and place attributes that contributed to a sense of home. Padgett [82] pointed out that having a roof over one’s head is necessary but not sufficient for having a “home” [84]. Anucha [77] further discussed “home as more than bricks and mortar.” The reviewed literature illustrated that PSH provided not only physical shelter and basic necessities, but a sense of home afforded by ontological security, safety, and trauma sensitivity. Home, ontological security, and safety related to individual resident experience of PSH while trauma sensitivity addressed how the built environment contributed to those experiences via design that responded to resident trauma.

#### 4.2.1. Sense of Home and Ontological Security

Housing provides shelter, but a “home” provides protection, security, safety, refuge, and centering; home is often described by comfort, privacy, familiarity, multiple layers of meaning, and a sense of self-expression, identity, responsibility, ownership, being “at one” within the setting, and an absence of mistreatment, alienation, and discomfort [70,85]. Eight articles addressed psychosocial benefits of home, including stigma, safety and security, privacy, and control [82]. Creating a sense of home was difficult because of the stigma and social exclusion that often resulted from PSH building design, physical condition, and neighborhood location [77,82]. Safety and security contributed to a sense of home [78,79], as did a sense of privacy [77,78,79]. PSH provided residents with a private space that allowed them to decide or control whether and how to engage with others [80,81] and determine who they permitted to enter their space [79]. A sense of home was also associated with having regular opportunities for social contact [83], a place to carry out daily routines [86], and “regular stuff” such as furniture, a microwave and dishwasher, air conditioning, a computer, and cable TV that made a house comfortable and feel like home [79]. Residents in Huffman’s study stated that being able to grow a garden made a place feel like home and not Skid Row [26]. PSH residents in McLane and Pable’s [27] study frequently commented on factors that contributed to a sense of home: location; aesthetics (cleanliness, acoustics, lighting quality, personalization, overall hominess); cleanliness and stigma as indicators of personal stake in or caring about the PSH facility; and personalization and creating a sense of ownership via decorative objects such as art.

A sense of home was closely related to ontological security [81,82], defined as the extent to which an environment supports constancy, the ability to complete daily routines, privacy and freedom from surveillance, control, and having a secure base for identity construction [81,82,86]. Characteristics of ontological security, including identity which is a psychosocial process that indirectly affects mental health [6], can be affected by the built environment. McLane and Pable [27] reported that PSH building design’s contrast with institutional settings can aid in post-homeless identity formation for the residents. Housing amenities, location, and nearby services also contributed to ontological security and being able to complete daily routines [82]. Most participants reported increases in ontological security after moving to PSH [82].

#### 4.2.2. Safe Haven

Five articles explicitly addressed PSH as a “safe haven” for residents, and 14 of 17 articles addressed safety and security (Section 4.3.3). Beyond safety and security, a safe haven provides a place of protection, refuge, and respite [87] relative to another previously occupied, less safe setting [88]. PSH can provide residents with a safe haven compared to living on the street or in an institutional setting. Safe haven characteristics include constancy, the ability to make choices, and a sense of decency, caring, and dignity [89]. Chan [79] described housing as a safe haven of peace and privacy where residents can choose to withdraw and spend time alone, but a safe haven could also be created through community and social connections [88]. Within the context of PSH, trauma sensitivity (Section 4.2.3) further contributed to creating a safe haven. Three studies [26,27,83] emphasized associations between safety and components of trauma-sensitive or trauma-informed design, including residential rather than institutional aesthetics, décor, hominess, natural light and views of nature, personalization, cleanliness, acoustics, and a welcoming yet secure lobby. In Padgett’s [82] study, homeless women, who experience higher rates of sexual and physical assault compared to homeless men [82], particularly expressed a need for the protective benefits of having their own apartment and “safe harbor.” Participants in Adame and colleagues’ [83] work described a desire for solitude, quiet and control, describing their apartments as a sanctuary from the outside world. Thus, residents reported that the built environment provided a safe haven across spatial scales including individual dwellings and the building within its surrounding location.

#### 4.2.3. Trauma Sensitivity, Trauma-Informed Care, and Trauma-Informed Design

While only five of the reviewed articles explicitly acknowledged the co-occurrence of long-term homelessness and trauma (Figure 2) and addressed connections between trauma and the PSH built environment, other studies implicitly addressed principles of trauma-sensitive design. Trauma sensitivity was defined as the extent to which a built environment supports the core principles of trauma-informed care. The Trauma Informed Care (TIC) framework is used by social and clinical service providers, including in PSH, and emphasizes rebuilding a sense of control and empowerment while providing physical, psychological, and emotional safety for those in recovery and their providers [90]. TIC principles include safety, trustworthiness, choice, collaboration, and empowerment [91]. Trauma-informed housing services are grounded in those principles, aim to maximize predictability, and respect privacy needs and healthy physical boundaries while providing opportunities for managing complex health issues, skill building, and social relationships essential to recovery [5]. However, as Huffman [26] concluded, *“missing from this framework is any sense of material space... the focus is on [trauma-informed care] practice [assessment, screening, treatment, resident services, programs, and case management], but not the physical context around those practices”* (p. 48). The recently coined term, “trauma-informed design,” refers to a developing design approach that emphasizes reducing or removing adverse environmental stimuli and stressors; providing multi-sensory environments, environmental supports for self-reliance and determination, and connectedness to nature; separation from others experiencing distress; reinforcing a sense of personal identity; and balancing opportunities for choice with safety and comfort [26,27,62,92].

Knight and colleagues [36] described trauma-sensitive built environments as clean, calm, controlled, self-contained, quiet, and new, or at least newly-renovated. They found that the degree to which SRO built environments were “trauma sensitive” was associated with greater stabilization among female PSH residents. Conversely, women in SRO environments that were not trauma-sensitive reported persistent fear, anxiety, sleep deprivation and hypervigilance, providing further justification that housing type, availability, and material conditions play a significant role in mental health [36]. Huffman [26] noted how the environment can respond to residents’ past traumatic experiences to avoid contributing to re-traumatizing or triggering events, such as prioritizing an aesthetic of deinstitutionalization and downplaying bureaucracy. The article also highlighted trauma-sensitive design aspects of the building intended to foster interaction, inclusion, and residential, rather than institutional, aesthetics and decor, such as an open-space layout, the inclusion of greenspace, and extensive use of glass walls in the common areas [26]. McLane and Pable [27] discussed trauma sensitivity within the context of the entire building, and specifically in shared common spaces (Section 4.4.3). Trauma informed care and design measures to protect safety and security, in particular, included clustering apartments in small groups with shared common space and restricting access to residents living within the cluster [27]. That policy, however, led to less use of shared spaces, residents feeling disempowered, and reduced opportunities for self-governance, interaction, and collaboration on shared goals [27]. Adame and colleagues [83] recognized the trauma of homelessness and pointed out that, for some PSH residents, the “*hypervigilance required for surviving life on the streets is hard to let go of, even when people have safe and secure housing situations*” (p. 1298); this hypervigilance led to residents avoiding common areas and retreating to individual dwellings. Hsu [73] found that social and physical disorder perceived by PSH residents in their neighborhood environment, which can be the same neighborhood in which they were homeless, can trigger memories of past victimization and traumatic experiences. Trauma-sensitive environments are particularly critical for this population when the location of the PSH is not trauma-sensitive.

### 4.3. Dwelling as a Vessel for Autonomous Daily Life

The second domain, “dwelling as a vessel for autonomous daily life” (Figure 2), addressed the importance of autonomy associated with housing that contributes to ontological security, privacy, control, and the ability to conduct daily routines [81,82]. Dwelling unit characteristics were reported as most important to participants [38] and were addressed by 13 papers. Dwelling unit type, size, occupancy, bathroom type, and access to storage, facilities, and nearby services, amenities, and transportation (Figure 2) were associated with numerous outcomes related to the improvements in freedom and autonomy often reported by PSH residents [21].

#### 4.3.1. Having One’s Own Space, Single Occupancy, and an Apartment versus a Room

Participants in seven studies reported benefits of having their “own space” [38,75,77,79,80,81,82]. With the exception of Wittman and colleagues’ review paper that suggested rooms in Sober Living facilities should be sized for more than one person in order to prevent relapse in substance abuse [71], single occupancy was explicitly preferred by residents [38,71,77,78,80]. Results from three studies further indicated that residents preferred independent apartments to rooms and SROs [74,75,77], and two additional studies emphasized that, in these preferred settings, interaction and use of shared space should be encouraged to prevent isolation, especially among residents in recovery [27,71]. The agency to choose and pursue goals related to control, autonomy, privacy, safety, territoriality, freedom, and ability to complete daily routines—that presumably accompany having a single occupancy dwelling unit, and an independent apartment more so than shared, SRO, and congregate settings—was associated with feelings of home, ontological security, identity construction, positive mental health, and well-being [79,81,82]. Dwelling unit safety and privacy related to ontological security [82] and having safe spaces for privacy, retreat, and isolation [79] were also important to residents. Having a safe space for and the agency to choose privacy was associated with feelings of home [79] and allowed residents to control interaction with others. The spatial hierarchy afforded by shared common spaces and semi-private and private dwelling spaces within independent apartments [80] support this control of social interaction. Independent apartment residents also reported significantly higher levels of housing choice and control and control over professional support when compared to participants living in group arrangements [74]. Moreover, independent apartments (and “en suite” bathrooms, Section 4.3.2) were not only preferred by and more beneficial to residents, they were more cost effective than SROs within one study, likely due to higher costs and complexities associated with renovation versus new construction [36].

Bigger rooms, more space, and more privacy were desired by participants [38,74,75,77]. When compared to independent apartments, SROs and smaller dwelling units were associated with lower mean housing quality scores, likely due to less privacy and fewer and shared amenities [38]. SRO residents also reported having less privacy [38] and the least choice due to small spaces, short-term leases, and transient neighbors [75]. Residents of shared housing settings similarly wanted more privacy [77] when compared to individual occupancy dwelling units. Participants in the reviewed studies clearly preferred single occupancy and apartments. However, more research is needed to understand whether these preferences represent all PSH residents and these preferences affect mental health.

#### 4.3.2. Preferences for Not Sharing a Bathroom

Studies revealed associations between having one’s own or “en suite” bathroom and positive outcomes, such as feelings of home in Chan’s study [79]. Knight and colleagues’ [36] qualitative analysis indicated that newly constructed, trauma-sensitive SROs with en suite bathrooms provided more resident choice, control, independence, safety, and security associated with better mental health outcomes when compared to old, poorly maintained SROs with shared bathrooms within the context of their study [36]. Although shared bathrooms were associated with a sense of safety in Burns and colleagues’ [78] study, they also triggered processes of territorial exclusion among older male participants with physical difficulties accessing shared bathrooms at night. Additional work is needed to evaluate mental health outcomes related to various bathroom types.

#### 4.3.3. Safety and Security

Considering the high rates of trauma, abuse, and victimization experienced by PSH residents [79], safety and security of PSH dwellings and buildings, regardless of surrounding neighborhood conditions, are critical and contribute to creating a sense of “home” and a safe haven [71,72]. Safety and security were addressed in 14 of 17 articles (Table S3, Supplemental Materials). Safety was discussed within the context of clustered apartment arrangements with controlled access [27]; floor level and being located above the ground floor [26,36]; aging in place and safety from harm due to falling and impaired mobility [78]; having a safe space [71,73,78,79,81]; having a sense of safety resulting from surveillance [78]; housing quality [38,72,74,75]; and safety from crime, drugs, and prostitution in the neighborhood [77]. Knight and colleagues [36] also revealed associations between perceived control and safety and having one’s own apartment among women residents. Although the terms “safety” and “security” were often used interchangeably by the reviewed studies, coding followed definitions as defined in Appendix B. Article definitions of security varied as the term was used as a synonym for safety and to refer to maintaining consistent possession of personal possessions, housing, and employment. Participants in Hsu’s [73] study discussed feeling safe and protected in PSH compared to the street due to decreased victimization, as well as PSH security measures such as having their own door to lock and the opportunity to grant entrance to individuals of their choosing; fences and signage; and visitor screening. Additionally, PSH residents noted the importance of other security factors that fostered a sense of safety, such as controlled entries to screen outsiders and keep “undesirable” people out [72,77]; security guards and compassionate police presence [77]; and security cameras and other forms of surveillance [36,73,78].

Location and neighborhood safety were also addressed by three studies and included in several of the housing quality measures. Brown and colleagues [72] found that PSH residents perceived the safety of their residence’s location as important and that it influenced their intent to stay in the housing. Hsu and colleagues [73] examined associations between perceptions and observer-rated measures of neighborhood safety and disorder among residents living in the Skid Row neighborhood versus its periphery. Although most participants reported increases in perceived and actual safety and security after transitioning to PSH, these perceptions were affected by the neighborhood environment such that increased physical and social disorder (e.g., more trash, malodors, and presence of homeless people observed in Skid Row) contributed to lower perceived safety and security. Residents of Skid Row reported spending more time within their housing facilities due to neighborhood safety concerns. Future, more conclusive findings about the increase in the amount of time residents spend inside PSH facilities have the potential to inform provider services, building siting, and PSH building design.

Although there is a critical need for safety and security within PSH, several studies emphasized the required balance with control and freedom from surveillance to achieve ontological security and a sense of home [81,82]. McLane and Pable [27] concluded that prioritizing safety and security over control reduced residents’ opportunities for self-governance and collaboration on mutual goals in their study. Efforts to protect resident safety via clustered apartments, surveillance, and controlled access to those clusters inhibited residents’ ability to feel empowered and interact with other residents. Burns and colleagues [78] further addressed the complex balance between safety, surveillance, privacy, and control, revealing that while surveillance enhanced residents’ feelings of safety, it encumbered their sense of privacy. PSH design and operation must ensure resident safety and security while simultaneously supporting residents’ ontological security.

#### 4.3.4. Housing (and Location) Quality

Housing quality can support or inhibit a sense of home and safe haven, as well as trauma sensitivity and autonomous daily life. Objective housing quality is rarely reported in studies of housing program interventions [38]. Five of seven articles that examined housing quality used standardized measures that included physical, ambient, spatial, and neighborhood properties, as well as place attributes. Studies focused on various measures of housing quality [38,74,75], along with housing type [74,75], housing satisfaction [72,75,76]; housing preferences [72,75], housing stability [38], and housing choice and control [74] within the context of mental health and psychological distress [76], treatment stage [75], quality of life [74,76], social support [75,76], relationship quality with staff [76], community adaptation of people with mental illness [74], treatment choice [76], and control over professional support [74]. Better housing, physical, and environmental quality were associated with increased housing stability [38]; satisfaction [72,76]); self-reported quality of life, adaptation to community living, and housing choice and control [74]. Table A3 (Appendix B) demonstrates variations in housing quality measures that varied in their depth and breadth; applicability to populations experiencing homelessness and behavioral health issues; inclusion of items related to the physical and social environment; and focus on spatial scales (room, building, and location). All studies focused on participant-reported housing quality, except for Adair and colleagues [38], who included both participant-reported and observer-rated measures of housing quality in their quasi-experimental study and disclosed all housing quality items included in the measure. Only selective housing items were disclosed in remaining measures, so only those that were articulated were coded. No studies addressing housing quality aimed to inform design, and the aggregated housing quality measures preclude drawing design conclusions.

Two of the five housing quality studies and three additional articles also addressed neighborhood condition and quality (aesthetics, garbage, trees/plants, malodors, cleanliness, presence of homeless people, sidewalk/street condition, building condition), noise, and safety. Better neighborhood quality—including increased safety, security, aesthetics and greenery and decreased stigma, noise, traffic, and garbage—was desired by participants [77] and associated with increased satisfaction [72]. As mentioned when discussing safety and security (Section 4.3.3), PSH location is important to consider as the context surrounding PSH can influence resident satisfaction with the built environment and other outcomes of interest.

#### 4.3.5. Access to Storage, Facilities, and Nearby Amenities

The importance of having a location to securely store belongings was discussed by participants in three studies relative to safety, security, agency, and feelings of home [73,78,79]; included in one housing quality measure associated with housing stability [38]; and mentioned by the review paper [71]. Being able to securely lock belongings and not lose them [78] as well as have “regular stuff” [79] contributed to safety, control, and the ability to carry out daily routines associated with ontological security. Similarly, individual or shared kitchen facilities that allowed individual residents to prepare their own meals and access to laundry facilities further supported autonomy, sense of home, ontological security, and satisfaction [38,81,82]. Beyond the building scale, ten studies (Table S3) addressed PSH building proximity and access to amenities and services (10), access to public transportation (6), and location and land use (5). Participants reported that this proximity and access (to, e.g., recreational activities, parks, retail locations and services, and transportation) contributed to housing satisfaction [72,76,77] and ontological security by being able to complete daily routines [82]. According to Wittman and colleagues [71], access can simultaneously facilitate and hinder program goals depending on proximity to supportive versus harmful services (e.g., available alcohol and other substances). Henwood and colleagues [93] found that PSH building siting both facilitated and inhibited social interaction for residents; residents able to maintain the positive social connections they formed during the time they were homeless benefitted, while those with harmful connections spent more time in dwelling units to avoid negative influences.

### 4.4. Shared Common Space and Sociality within Single-Site PSH

#### 4.4.1. Setting Facilitates Community

The third domain focused on the place attribute, sociality, defined as the degree to which an environment facilitates or inhibits social interaction [68]. Five of the reviewed studies addressed sociality and shared common areas within PSH relevant to the formation of social support. In addition to individual recovery and skill building, single-site PSH models focus on assisting residents with building socially supportive relationships and integrating with other PSH residents and the surrounding community [18]. Adame and colleagues [83] found that mutual support, care, feeling comfortable in shared spaces, and respecting each other’s possessions and boundaries was necessary to care for and share common space with others. PSH can facilitate community via careful balance of the privacy afforded by dwelling units and the social interaction encouraged by shared common areas [27,71]. Single-site PSH, in particular, can encourage formal and informal social interaction within shared common spaces that contribute to the formation of social support, a mediating mechanism of the built environment and mental health relation [6]. Three studies focused on common areas reported that the relationship between residents was important to their sense of community, as well as their relationships with staff [26,27,83]. Adame and colleagues [83] noted that formal and informal gatherings and events in shared common areas were particularly important for building a sense of community within the PSH facility. Chan [79] added that choice of activity and social interaction contributed to residents’ common area satisfaction. Residents, however, must be able to control desired levels of interaction and privacy rather than be forced to interact or be alone due to spatial or operational constraints which can result in social withdrawal and isolation [34,94,95]. The importance of the interaction between social and physical design factors surrounding sociality and shared common areas, as Wittman and colleagues’ stressed [71], is reflected in the following sections concerning PSH resident experiences of shared common spaces and design factors explored by several studies.

#### 4.4.2. Promising and Contested Shared Common Spaces

While Adame and colleagues [83] indicated that the built environment provides opportunities for events and activities that, when regularly held, help to build community, create a sense of stability, and increase familiarity and trust among residents, Huffman [26] emphasized that common space is both promising and contested. Based on an investigation of shared common areas as high- and low-stakes environments, tensions arose between residents when common areas had resources that could be depleted, such as in community gardens growing vegetables; residents who participated in the cultivation of the garden tended to exclude those who did not. Common areas with non-scarce resources, such as sitting rooms, were less contested and used less [26]. Burns and colleagues [78] found that tension resulted from interactions between design and housing rules: while the cafeteria facilitated social interaction more than residents eating alone in their rooms, the forced social contact due to prohibiting in-room refrigerators and absent cooking facilities in dwelling units was not always welcome. A shared television room in Burns and colleagues’ [78] study also contributed to territorial exclusion and spatial segregation resulting from language differences that led to a contested television room. Only members of one language group could gather and watch TV at a time, excluding the other. These examples suggested that design can promote or inhibit the flexibility required to accommodate different social interaction needs and varying levels of privacy and interaction, as well as tend to PSH resident needs relating to trauma sensitivity and safety.

#### 4.4.3. Sociality and Trauma Sensitivity

As Huffman [26] stated, although shared common spaces cannot address all social issues, common spaces intentionally designed to respond to social needs and trauma can positively affect well-being and provide a place for interaction [27]. Huffman [26] and McLane and Pable [27] aimed to identify design characteristics that are not only trauma-sensitive, but also promote and enhance social interaction and sense of community among PSH residents. In their case study of two facilities, McLane and Pable [27] found that common space was reported by both staff and residents as important, yet underused. They found that common space location, wayfinding, and visual and physical access were the most important design characteristics of successful common spaces. Common space location relating to placement along circulation pathways, measured by methods known as space syntax [48], affected visibility and use. They explained that the “deeper” (farther from an entrance or well-traversed pathway) a space was located in a building, the more difficult the space was to find and access. This additional effort, even if perceived, reduced the likelihood of PSH residents using that space. Similarly, the interior visibility of a shared space, or a lack thereof, affected use. McLane and Pable [27] posited that residents likely felt more comfortable engaging with a space if they were able to first see and assess who was in it from a safe psychological distance. Without an opportunity to visually evaluate a space before deciding whether or not to engage, residents, especially those who have experienced trauma, may feel vulnerable and avoid approaching that space [27]. This finding aligned with literature suggesting that persons with a history of stress, violence, and other trauma associated with homelessness may feel vulnerable to being seen or spoken to by others [51]. McLane and Pable described Appleton’s [96] prospect-refuge theory as an explanation for this behavior: people prefer environments with the ability to observe (prospect) from an enclosed, safe space (refuge) without being seen by others [27].

#### 4.4.4. Sociality by Design

After location, visibility, and access, McLane and Pable [27] concluded that aesthetics, hominess (views, personalization, cleanliness, acoustics, lighting, daylight), and size (large enough for multiple activity zones) were the most important design factors of shared common spaces (common areas, bathrooms, cafeterias, rec rooms, TV rooms, and smoking areas). The size [27,71,78] and quantity [26] of shared common spaces available to residents created a hierarchy of public to private space that is discussed in other residential literature as associated with balancing privacy and social interaction and avoiding isolation and withdrawal [45,97]. This hierarchy offered residents flexibility and choice within in their environment and control over their desired levels of interaction. McLane and Pable [27] also described “spatial choice,” or the ability to choose between different common areas and pathways relative to shared common areas. Burns and colleagues [78] noted that space and the built environment can provide or prohibit choice in, for example, interacting with others in a congregate living setting via offering shared or individual cooking and dining facilities that affect where, when, and what residents eat; this agency and spatial choice can mitigate territorial exclusion. In addition to having multiple shared common spaces of varying sizes, the need for varying levels of privacy was highlighted. In their case study, McLane and Pable [27] found that common spaces were typically most often used by one person or small groups of two to three people at a given time. The creation of distinct “functional zones” separated by pathways, low partitions, plants, or furniture with higher backs can create a sense of privacy to encourage and increase use of common areas [27]. Wittman and colleagues [71] added that a variety of spatial arrangements—related to functional zones—that promote varying levels of privacy and interaction should include socio-petal spatial layouts that promote interaction. Offering residents a choice of public, semi-public, and semi-private common areas promotes balance with private dwelling units for personal time, sleeping, and hygiene.

## 5. Discussion

### 5.1. Interpretation and Implications of Findings

This integrative review identified architectural design and built environment characteristics relevant to PSH residents’ mental health and well-being. Three domains were identified related to the PSH experience of home and trauma-informed design; housing types, quality, and location; and the design and placement of shared common spaces. Collectively, the appearance of built environment factors in the 17 reviewed articles suggested that the PSH built environment—at multiple spatial scales—matters and is worthy of future study. In their review, Wittman and colleagues [71] described the importance of recognizing the role of the built environment in social programs and services addressing homelessness and behavioral health issues and asserted that the interaction between the setting and desired outcomes must be considered. Relevant built environment findings in the other 16 articles reviewed supported these statements and aligned with existing built environment and mental health literature. Results from this review revealed that “home” is more than material housing, and that housing quality and design factors likely contribute to mental health and well-being. Success in addressing homelessness is often based on housing counts and the ratio of housing units or beds provided to the estimated number of people experiencing homelessness. The reviewed studies, however, revealed contributions of built environment quality and the process of making a house a “home” to recovery and mental health. The emphasis on housing quantity is also reflected in the reviewed literature’s exploration of dwelling units more than shared common spaces, yet social relationships are critical to PSH resident recovery [18]. With robust future research and more conclusive findings, these built environment contributions can inform the development of evidence-based design guidelines and have significant implications for design practice, program evaluation, research, and policy addressing homelessness.

Results of this review converged with existing built environment and mental health literature, offering further justification that the built environment matters and that future PSH research and design can be informed by this prior work. Findings from this review paralleled other built environment and environmental psychology literature that explored a sense of home and ontological security, identity, and a safe haven; autonomy, privacy, safety, and control associated with residential settings; and balancing privacy with opportunities for social interaction (see Background). Each of these place attributes requires a balance of social and physical factors to achieve, including environmental quality, cleanliness, preferred dwelling types, layout, and residential rather than institutional aesthetics. This review further demonstrated that place attributes defined by environmental psychology offer a useful approach to describing built environment and experiential qualities that likely affect PSH resident health and well-being [35].

Trauma sensitivity and trauma-informed design discussed in the reviewed articles offered new directions for and applications of environmental psychology to PSH. Designers and environmental psychologists, among other disciplines and professionals, have examined and emphasized the built environment’s role in physical health, mental health, and social well-being, yet the same principles are not always incorporated into PSH research and design [27]. These principles, along with trauma sensitivity and trauma-informed design, can be researched within the context of and applied to PSH design to improve mental health and other outcomes of interest.

In addition to applying an environmental psychology framework to review the PSH built environment, this review adds a built environment perspective to the PSH literature. Only two of the 17 reviewed articles were design driven [27,71] and only four included any building or facility description at all [e.g., number of units and floor levels, building type (high-rise, low-rise, duplex, brick apartments, historic brownstones, etc.), ceiling height and floor plan layout, available shared spaces] [26,27,38,79]. In this review, spatial scales of room (dwelling unit and shared common space), building, and location; built environment properties (ambient, physical, and spatial); and place attributes provided a framework for identifying, describing, and discriminating between relevant built environment factors. This framework can inform future PSH research, program and facility evaluation, and design. Examining interactions between spatial scales and physical and social factors on mental health and other outcomes also offers directions for future research. Moreover, review results indicated that both subjective (e.g., housing satisfaction and preferences) and objective (e.g., observer-rated housing quality) measures of the built environment influence resident outcomes. For example, residents who lived in their preferred housing type reported significantly greater choice over housing and activities than residents not living in their preferred housing type [75]. These findings were consistent with other work that found that perceptions of the built environment, as well as objective measures, matter and are often better indicators of satisfaction and mental health outcomes [35].

Although there was an absence of architecture and design in the reviewed literature, influences of and implications for architecture and design were present. For example, preferences for independent apartments (Section 4.3.1) and en suite bathrooms (Section 4.3.2), rather than SROs or congregate settings with shared kitchens and baths, and the need for a variety of appropriately located shared common spaces (Section 4.4.3) have significant implications for architecture and building design. Floor plan arrangements and building footprints, site requirements, and construction costs vary based on housing, bathroom, and shared space quantity and type. Considering resident need for control and choice, the often non-linear and fluid nature of recovery, and varied preparedness to transition to an independent apartment from living on the street, future research is needed to determine the effects of different facility designs on resident mental health and well-being, service delivery, and costs. These considerations are especially relevant as PSH resident preferences for independent apartments and needs for shared common space align with a market-rate apartment complex trend in the U.S. to increase the amount of shared common and recreational space available to residents to provide opportunities for social interaction. These apartment buildings can potentially serve as models for PSH as they intend to promote spatial choice and autonomy via a variety of dwelling types and shared common spaces that balance opportunities for privacy and social interaction while discouraging isolation [78]. Furthermore, several U.S. states now construct more independent apartment buildings (e.g., Massachusetts) to address homelessness for two reasons. First, some states (e.g., Indiana) do not permit SROs. Second, buildings with apartments rather than SROs with dormitory-style rooms can be more easily converted into market-rate rental apartment buildings because each unit has its own bathroom and kitchen compared to SROs or congregate settings with shared bathrooms and kitchens. Effects of these apartment buildings on mental health, however, have not been extensively evaluated in market-rate or PSH settings. Evidence-based design guidelines are needed to inform the design of dwelling units, shared spaces, and buildings to support PSH resident recovery and program goals, as well as consider adaptability for future needs.

### 5.2. Strengths and Limitations

To our knowledge, this study is the first integrative review focused on PSH architecture, design, and built environment characteristics relevant to mental health. A broad and systematic search across multiple disciplines that followed PRISMA guidelines was conducted to answer the research question. Whittemore and Knafl’s [60] five-part integrative review methodology guided problem identification, the literature search, data evaluation, data analysis, and result presentation. The review team included researchers with experience in quantitative and qualitative research, architectural practice, and working with PSH residents and facilities as architects and researchers. The integrative review process included both quantitative and qualitative studies in the iterative analyses. However, the nature of integrating findings from qualitative, quantitative, and mixed-methods studies inevitably required some degree of subjectivity and interpretation. The power of the four quantitative studies was also diminished due to the integration process [30], and review results were limited to an integrative and descriptive rather than conclusive summation. Results did not represent a comprehensive or conclusive list of built environment factors related to mental health. Moreover, several relevant topics from the existing built environment and health literature were noticeably absent from or minimally appeared in the reviewed studies, as described in Section 5.3.2.

The systematic approach to the integrative review, including the inclusion and exclusion criteria, was a strength as well as a limitation. The review focused on single adults living in PSH in the U.S. and Canada, so the review had population and geographical biases. Studies about shelter and transitional housing, residential settings for the elderly, and other literature where service models and supportive and supported housing definitions differ (e.g., Europe and Scandinavia), were excluded, but could still be relevant to understanding influences of the built environment on mental health. Other relevant studies may have been excluded due to inconsistencies in the literature, including the interchangeable use of “supported” and “supportive” housing, incomplete reporting of data collection measures (e.g., interview questions and questionnaire items), and unspecified housing type and service model. Finally, the qualitative studies reviewed illuminated why or how the built environment may affect mental health and other outcomes according to often non-representative groups of participants so more representative work is needed. Quantitative studies focused on the built environment that investigate those experiences and identify specific built environment factors contributing to outcomes of interest are desperately needed to generate evidence-based and generalizable design guidelines and a body of rigorous evidence required to inform policy.

### 5.3. Directions for Future PSH Research

Despite widespread implementation of PSH and Housing First in the U.S. and Canada, research on the design and built environment of PSH facilities is lacking. A large body of literature assesses the PSH model, but rarely the space and place in which those programs occur. McLane and Pable [27] stated that, *“architecture as a discipline has pivoted toward more human-centered approaches, with the individual’s dignity and well-being at center stage”* (p. 1); however, little human-centered or evidence-based guidance exists to inform PSH architects and designers [26,27,35,71]. Beyond Adair and colleague’s [38] comprehensive, quasi-experimental housing quality study, McLane and Pable’s [27] common area design characteristics, and Anucha’s [77] brief mention regarding the size and number of people recommended to share common spaces, no evidence-based design guidance was identified in this review. The following sections describe how future research can increase the quantity and quality of PSH built environment and mental health work and suggest topics for inclusion that were largely missing from the reviewed literature.

#### 5.3.1. Increasing the Quantity and Quality of Research

Future design-driven exploration of discrete architectural environments is needed to generate more conclusive results concerning the PSH built environment and mental health [27]. Exploring direct effects of and interactions between the PSH built environment and residents’ mental health is essential to determining the strength of these relations. Longitudinal studies of representative PSH facilities and populations that focus on specific design features, such as building form, surrounding neighborhood characteristics, views of and proximity to nature, floor levels, layout, unit quantity, ceiling height, daylight and window design, or décor (see also 5.3.2), and mental health outcomes are required to identify main effects of these traits on mental health. An examination of interactions between these design features and resident and staff activities and perceptions is also necessary. Moreover, comprehensive, reliable, and valid built environment assessment tools specific to PSH must also be developed and tested to explore the cumulative effects of dwelling, shared common area, and building design nuances in context. Such systematic tools enable evaluation of larger and representative samples of facilities across regions and can often be analyzed using secondary mental health datasets. Inclusion of collaborators with architectural or design experience on multidisciplinary quantitative and qualitative research teams can inform study design, selection of design features for evaluation, and interview prompts and follow-up questions concerning design features. Additionally, future qualitative studies focused on the built environment and mental health that include representative samples of a facility or region are needed. Rigorous mixed-methods studies comparing objective and subjective measures are also valuable and necessary.

Future quantitative PSH work can benefit by addressing common methodological challenges affecting existing housing research and working to improve study rigor and generalizability. Identifying and including moderating factors and mediating mechanisms that affect the built environment and mental health relation [6,34] and contribute to causal pathways, as well as achieving high internal, external, and construct validity is critical to building a strong evidence base capable of informing PSH operations, design guidelines and practice, and policy. Establishing causal direction between housing and mental health is particularly challenging as consequences of homelessness contribute to and exacerbate mental illness, and the social and financial consequences of mental illness contribute to homelessness. Longitudinal cohort studies are needed to identify the pathways to and risk factors for homelessness, as well as to assess housing interventions that integrate treatment for mental illness and substance abuse [7]. Regarding internal validity, the majority of identified studies in this review were cross-sectional. Only one study [38] was quasi-experimental as random sampling and assignment are rare in housing studies and preclude identification of causal pathways. Longitudinal studies can generate stronger evidence needed to establish causal relations, and well-designed cross-sectional studies that statistically control for confounding variables can improve internal validity [98]. Cohort studies that examine effects of the built environment related to various behavioral health diagnoses may also be useful. It must be noted, however, that these alternative study designs with the potential to establish causal pathways are not always effective [98], which makes determining whether the built environment is a causal or correlational factor of mental health outcomes difficult.

Generalizability is another challenge in housing studies. Like most cross-sectional and even longitudinal housing research, the generalizability of PSH and mental health research is limited. Applicability of findings to other building designs, locations, populations, age and demographic groups, and cultures requires more rigorous study designs and sampling techniques, which may be difficult to achieve. Sampling, recruiting, and retention complications among the homeless population as well as the inability to randomly assign people to housing limit external validity. Translation of findings to inform design guidelines, design practice, PSH service delivery, and policy related to the built environment, however, requires generalizability which presents additional questions that must be answered by rigorous research. Future research focused on the built environment and mental health can explore how large existing datasets, such as national health insurance claims or data informing precision health research, might be leveraged. Including larger sample sizes, more buildings, and buildings with varying design and spatial layouts can enable the use of more advanced statistical models [27] necessary for improvement of study rigor and generalizability.

With respect to construct validity, most mental health outcome measures were validated in these studies while built environment outcome measures ranged from validated instruments to self-reports and anecdotal responses to interview questions. How to measure individual and collective aspects of the built environment within the context of PSH and mental health requires additional and more rigorous investigation. Once direct effects and valid measures are complete, more complex investigations can begin to quantitatively and qualitatively explore interactions between architectural design and explicit and implicit PSH policies and social hierarchical factors [27].

#### 5.3.2. Topics for Future Research

As previously mentioned, several topics relevant to the built environment and mental health were absent from the reviewed literature and should be explored in future research, including several place attributes: legibility and wayfinding, accessibility, adaptability, aging in place, sensory stimulation, and restoration and associated design approaches and features. Legibility and wayfinding refer to a building’s layout and how occupants find their way to destinations (see Appendix A, Table A2 for complete definitions). Buildings that are difficult to navigate can contribute to stress and diminished perceptions of safety [99], which can interfere with a sense of home and safe haven. McLane and Pable [27] mentioned wayfinding with respect to locating shared common spaces in PSH, but no other studies addressed wayfinding or legibility. Similarly, issues relating to mobility and accessibility were only mentioned once (older male participants in the Burns and colleagues [78] study struggled with accessing bathrooms “down the hall”), which was surprising since PSH often houses people with physical and cognitive disabilities [1,19]. In the U.S., architecture contributes to accessibility via required compliance with the Americans with Disabilities Act, but design can exceed these minimum thresholds for accessibility via an approach known as Universal Design. Universal Design is a dynamic design approach that aims to create products and environments that are usable by the widest possible range of people regardless of body type, ability, or situation [100].

Aging in place— the ability to live in one’s preferred home and community safely, independently, and comfortably regardless of age, ability level, or income [78]—was also only mentioned once in the reviewed literature. Built environment approaches that support aging in place and accessibility, including universal design, tend to provide more supportive environments for all and may be particularly beneficial for aging PSH residents [101]. Furthermore, the place attribute, adaptability, can support accessibility and aging in place [85], but refers more generally to the ability of spaces to support multiple uses [68]. Adaptability was not discussed in any of the reviewed studies, but could address the need for “functional zones” in common areas [27] and a variety of spaces to support a balance of privacy and social interaction.

Another attribute of place, restoration, has great potential to benefit PSH residents. Only two articles mentioned restoration via describing dwelling units as a place to restore and retreat [79,83], but no mention of restorative design elements were found in the reviewed literature. Restoration (defined in Appendix A, Table A2), especially related to time spent in and views of nature, has been associated with cognitive functioning, recovery from stress, and mental health outcomes [28,35,102]. Healing elements of therapeutic and restorative design [28,103], including biophilic design that emulates aspects of nature in the built environment [104], offer research questions and guidance for PSH practitioners, researchers, and designers.

Finally, this integrative review focused on single adults in the U.S. and Canada, so investigations of the built environment and mental health within PSH facilities for families and youth and in other regions are needed. Additional research is also required to establish how the built environment affects specific subgroups within the homeless population. For example, persons with different mental health diagnoses may respond differently to spatial layouts intended to promote visibility and varying levels of social interaction and privacy [105]. Moreover, built environment experiences can vary by age, race, sexual orientation, gender identity, and intersections of these resident identities [83,106]. Transgender and gender non-conforming adults experiencing homelessness, especially, encounter unique challenges particularly regarding safety [107]. Bullying and antisocial tendencies related to shared common areas must not be overlooked [27]. Inclusive and representative research is needed to identify built environment characteristics that support each PSH resident.

## 6. Conclusions

Overall, integrative review results suggested that influences of the PSH built environment on resident mental health are worthy of further research. As Wittman and colleagues [71] pointed out: ***“****Architectural characteristics… need to be viewed as a vital partner in service delivery rather than a neutral container that simply provides shelter*” (p. 162). The physical surroundings of people formerly experiencing homelessness influence recovery, especially from trauma [27,70]. The needs of this population must be at the center of both social policies and programs *and* design solutions [27,70,108]. While PSH built environment and mental health work is nascent, largely exploratory, and faces methodological challenges, the fairly recent studies reviewed provide guidance for future research and design. Future work should balance increasing the amount of rigorous and large-scale quantitative studies with mixed-methods and qualitative work needed to make inferences about quantitative indicators, as well as framing study design with translation of research to practice. Additional studies are necessary to identify design characteristics that both support and hinder resident mental health, address knowledge gaps, and inform evidence-based design guidelines to optimize environments for PSH residents. Key questions for future PSH research include determining the optimal built environment for PSH residents and identifying specific built environment characteristics with the greatest return on investment to prioritize often limited resources. This emerging area of research has the potential to influence and advance PSH design practice, service delivery, and policy addressing homelessness, behavioral health, and health equity.

## Figures and Tables

**Table 1:** Databases and search terms.

Search Concepts	Search Terms ^1^
Supportive(ed) Housing ^2^	Housing, residential
Mental Health	Mental health, mental illness/es, mental disorder/s, mental well-being, psychological illness/es, behavioral health, psychiatric disabilities, loneliness, trauma, psychological health
Architecture/Design	Built environment, interior design, architecture, physical environment, environmental design, environment design, design attributes, design features, architectural, spatial characteristics, design characteristics, safety, security, surveillance, wayfinding, territoriality, crowding, privacy, housing quality, environment quality, environmental quality
Excluded	Children, older adults, later life, elderly, aged, disabled, older people, care facilities, board home, care home, nursing home, nursing homes, city, cities, urban, eating disorder, eating disorders, mental retardation, prison, prisons, jail, jails, HIV, AIDS, refugee, refugees, asylum, youth, adolescent, adolescents, workplace, workplaces, COVID-19

^1^ See Table S1 for complete search syntax. Search terms were applied to each of 9 databases (for exceptions, see Table S1): CINAHL Health Source, JSTOR, ProQuest, PsycInfo, PubMed, Scopus, Social Service Abstracts, and Web of Science. ^2^ Search terms relating to homelessness were not applied to avoid narrowing search results.

**Table 2:** Built environment relevance and methodological rigor ratings.

a. Built Environment Relevance	b. Methodological Rigor Ratings [60]
**Design driven** (DD): Studies contained independent and/or dependent variables addressing built environment factors associated with design (e.g., size, location, adjacency); study aims included evaluating and/or informing architectural design. **Built environment focused** (BE): Studies included BE independent and/or dependent variables (e.g., housing quality), without aims to evaluate or inform architectural design. **Inductive** (IN): Qualitative studies that indirectly addressed built environment factors in research questions (e.g., aspects of “home”) and yielded findings or responses relevant to design. **Mentions** (ME): Qualitative studies that did not include the built environment in the research design, but findings (i.e., participant responses) frequently “mentioned” built environment factors consistent with studies in the other three relevance categories.	**Quantitative**: Rigor was rated on a three-point scale (high, medium, or low) based on research design (e.g., longitudinal or cross-sectional, control or comparison group), sampling technique (random,, purposeful, or convenience) and size, data collection methods and measures (e.g., single or multiple, self-reported and/or objective, tested or newly created) including reported psychometrics (e.g., reliability and validity), and analysis methods (advanced statistical analysis vs. descriptive statistics) and reported measures of effect size with results. **Qualitative**: Rigor was rated on a three-point scale (high, medium, or low) based on the research design (cross-sectional, repeated measures, or longitudinal), sampling technique (purposeful or convenience), data collection methods and measures (e.g., cited instrument, accuracy check), and coding and analysis techniques (e.g., iterative, multiple coders, cited method, end-stage validation). **Mixed Methods**: After rating the quantitative and qualitative components of mixed-methods studies according to the rigor definitions, an overall rigor rating was discussed by researchers based on whether the study was primarily quantitative or qualitative. **Review**: Rigor was assessed based on reported search processes and analyses procedures (neither were reported in the single review).

**Table 3:** General characteristics of the reviewed literature.

Citation Location	Purpose ^1^	Research Design ^2^ STUDY TYPE (Research Design) - Data Sources (See *#) - Sample size—Sampling Approach - Age (Years) | Participant Details | Time Housed	Rigor	Housing ^3^ - Type/Program - Site Approach - Dwelling Type	Scale ^4^ Dwelling, Room, Building, Location			
					D	R	B	L
**Relevance: Design Driven (2)**								
McLane et al., 2020 [27] U.S. (Tallahassee); UK (Southampton)	Recorded and explored socio-spatial and design factors, policies and programming, and resident perceptions of shared community gathering space location, design, and use in two PSH facilities with the aim of presenting new analysis methods and improving future shared spaces.	MIXED (Dual case study; CS) - Space syntax, questionnaires, open-ended interviews, photography - n = 38 residents and staff—Convenience [12 staff and 23 residents (6 not formerly homeless)] 28 surveys, 18 interviews - Participant details and time housed not specified	Medium QUAN-Low QUAL-Med	- PSH - SS - Ind apt, Cong		•	•	
Wittman et al., 2017 [71] U.S. (review article)	Provided an overview of Housing First (HF) and Sober Living Housing (SLH) models and recommendations for practice based on an approach to architectural planning that emphasized the interaction between settings and operations on resident experiences.	REVIEW (not specified) - Source types: Existing literature; authors’ own research and practice providing residential substance abuse and mental health services; architectural planning papers that emphasize the interaction of settings and operations to achieve service goals; and authors’ involvement in national organization forums about housing models for homeless persons	Low	- HF, SL - SS, Scat - Varies	•	•	•	•
**Relevance: BE Focus (8)**								
Adair et al., 2016 [38] Canada (Moncton, Montreal, Toronto, Winnipeg, Vancouver)	Assessed housing quality in Housing First (HF) and Treatment as Usual (TAU) facilities, examined differences between participants in each group, and studied associations between housing quality and housing stability.	QUANTITATIVE (Quasi-Experimental, Longitudinal—2 yrs) - Observer ratings *, publicly available data, interview questions - n = 438 adults in 4 cities—RS (TAU = 228, HF = 204) - 42 | 63% female | At least 2 months housed	High	- HF, TAU - SS, Scat, Priv Mkt - Cong, SRO, Apt (ind, shrd)	•		•	•
Brown et al., 2015 [72] U.S. (Seattle)	Explored perceptions of housing and neighborhood environments and associations with satisfaction (high/low desire to stay) among single-site Housing First residents via the Housing Environment Survey.	MIXED (2-group comparison, CS) - Participant ratings *, open-ended interviews - n = 33 adults (30 interviews)—Convenience - 43 | 72% male | 61% White 82% psychotic disorder | 61% substance use disorder | 3 yrs housed (SD = 313 days), on average	Low QUAN-Med QUAL-Low	- HF, PSH - SS - Apt (75 Ind)	•	•		•
Hsu et al., 2016 [73] U.S. (Los Angeles)	Examined perceptions of safety and security among residents living in and surrounding the Skid Row area of Los Angeles and how those perceptions correlated with objective measures of neighborhood environment.	MIXED (Explanatory sequential; CS) - Semi-structured interviews, block-based neighborhood characteristic ratings - n = 24 adults (long-term homelessness)—Criterion sampling - 50 | 67% male | 71% Black, 0% White | 54% experienced victimization | 3 months housed	Low QUAN-Low QUAL-Med	- HF, PSH - SS (8 projects) - Not specified	•		•	•
Huffman, 2018 [26] U.S. (Los Angeles)	Investigated the connection between PSH social spaces, participation, and community based on resident experiences in a housing organization on Skid Row in Los Angeles, California.	QUALITATIVE (Case study, CS) - Pragmatic field work (1.5 yrs), semi-structured interviews, primary cycle analysis ^#^ - n = 26 of 100 residents—Convenience - 60 (avg. for all 100 residents) | Time housed not specified	High	- HF, PSH - SS - Ind apt		•		
Knight et al., 2014 [36] U.S. (San Francisco)	Explored how SROs can operate as “mental health risk environments” in which macro-structural factors (housing policies shaping the built environment) interact with meso-level factors (social relations within SROs) and micro-level, behavioral coping strategies to affect women’s mental health.	QUALITATIVE (Longitudinal, Ethnography—4 yrs) ^#^ - Interviews (baseline, 12, 18 months), photo-ethnographic study of SRO rooms - n = 30 women—Purposeful from larger study - Co-occurring mental health and substance abuse issues | Extensive histories of victimization | Time housed not specified	High	- Varies - SS - SRO hotel	•			•
Nelson et al., 2007 [74] Canada (Toronto, Hamilton, Ottawa)	Examined whether consumer choice and control over housing, support, and housing quality contributed to self-reported quality of life and adaptation to community living among people with mental illness, and whether individual apartments provided more choice and control than group living arrangements.	QUANTITATIVE (Repeated measures, CS) * - Structured interview + 9-month follow-up - n = 130 adults (90 = follow-up interviews)—Convenience (97 = independent apartments, 33 = congregate housing) - 41 | 58% male | 209 days (15–2042 days) housed, on average	Med	- Supported - SS, Scat - Apt (ind, shrd)	•		•	
Tsai et al., 2010 [75] U.S. (Chicago)	Examined whether housing preferences differed between substance abuse treatment stages, whether dual-diagnoses consumers who prefer certain housing types preferred certain characteristics, and whether consumers residing in different housing types reported differences in choice, social support, and housing satisfaction.	QUANTITATIVE (Group comparison, CS) - Questionnaires * - n = 103 dual-diagnoses consumers—Convenience (65 supervised, 38 community, 22 ind apt, 11 SRO, 5 family) - 45 | 75% male | 57% Black | Time housed not specified	Med	- Not specified - Not specified - Supervised, Ind apt, SRO	•		•	•
Tsai et al., 2012 [76] U.S. (11 sites, locations not specified)	Identified primary domains of housing satisfaction (HS), tracked HS over time, and assessed relations between HS and subjective and functional outcomes.	QUANTITATIVE (Longitudinal: quarterly for 2 yrs) - Structured interviews, questionnaires - n = 756 (11 sites)—Criterion sampling - 45 | 75% male | 62% Minority | Time housed varied	High	- HF, PSH - Varies - Not specified			•	•
**Relevance: Inductive (6)**								
Anucha, 2005 [77] Canada (Toronto)	Explored the needs of the formerly homeless, from their perspective, and how housing, neighborhood, and community can meet their needs more effectively to avoid a return to homelessness.	QUALITATIVE (Exploratory, CS) - Open-ended interviews, thematic analysis - n = 106 “hard-to-house adults”—Convenience - 45 | 60% male | 68% White | ≥3 months housed	Low	- HF (2 programs) - SS (2 buildings) - Cong.	•		•	•
Burns et al., 2020 [78] Canada (Montreal)	Explored everyday experiences of formerly homeless older men residing in single-site PSH based on the concepts of home and social exclusion.	QUALITATIVE (Const. grounded theory, CS) - Semi-structured/in-depth interviews - n = 10 males—Provider recruited - 55–70 | 90% substance abuse | Time housed not specified	High	- PSH, SL - SS - SRO	•	•		
Chan, 2020 [79] U.S. (Boston, Cambridge)	Explored what makes supportive housing feel like “home” for individuals who were once homeless related to constructing new, non-homeless identities, social isolation, and community integration.	QUALITATIVE (Repeated measures, CS) - Drawing, 2 semi-structured interviews ^#^ - n = 37 adults—Convenience (15 ind apt, 17 SRO, 5 cong apt) - 52 | 54% female | 51% White | 92% physical disability | - 67% psychological disability | 4 yrs housed (1 month–16 yrs), on average	Med	- HF, PSH - SS, Scat - SRO, Ind apt, Cong apt with 1–2 roommates	•	•	•	
Henwood et al., 2018a [80] U.S. (Los Angeles)	Considered how contextual factors generate or reduce risk for substance use among adults who recently moved into PSH.	QUALITATIVE (Case summary matrix, CS) - Ethnographic shadowing (3.5 h) - n = 27 adults—Purposeful (risk profiles) from a larger study - 55 | 59% male | 59% Black | 22–59% MH diagnoses | Time housed not specified	Med	- HF, PSH - SS, Scat - Ind apt	•			•
Henwood et al., 2018b [81] U.S. (Los Angeles)	Used ontological security (well-being rooted in a sense of constancy in the social and material environment) as a sensitizing framework to examine the perspectives and experiences of young adult PSH residents.	QUALITATIVE (Grounded theory, CS) - Semi-structured interviews ^#^ - n = 29 young adults—Convenience - 23 (18–25) | 62% male | 14% White | 68% heterosexual | 18 months housed, on average	Med	- PSH - SS (4 buildings) - Ind apt	•			
Padgett, 2007 [82] U.S. (New York City)	Explored how study participants who obtained independent housing experience, enact and describe having a “home” and to what extent their experiences reflect markers of ontological security.	QUALITATIVE (Grounded theory, CS) - 2 life-history interviews (2nd for accuracy) with control group comparison ^#^ - n = 39 participants with DSM Axis 1 disorder—Purposeful - (HF = 21, TF = 18) - 48 | 67% male | 41% White | 90% co-occurring substance use | Time housed varied	High	- HF, TF, Supervised - Ind apt, rooms in transitional “treatment housing”	•		•	•
**Relevance: Mentions (1)**								
Adame et al., 2020 [83] U.S. (Seattle)	Interviewed residents of a Housing First organization about their experiences of community and gathered suggestions for improving community building efforts.	QUALITATIVE (Exploratory, CS) - Focus groups, interviews, thematic analysis ^#^ - n = 38 residents—Convenience - 56 | 66% male | 47% White | 4 yrs, avg. (1 month–23 yrs)	Med	- HF, PSH - SS (8 buildings) - Not specified (SRO or ind)		•		•
Table Notes and Abbreviations * = Psychometrics were reported for the quantitative measure(s) used to collect independent and/or dependent variables. ^#^ = Qualitative methods included procedures that addressed rigor in data collection, coding, and/or analysis. ^1^ = “Purpose” column: HF = Housing First; HQ = housing quality; PSH = permanent supportive housing; TF = Treatment First; SLH = Sober Living Housing; TAU = Treatment as Usual		^2^ = “Research Design” column: MIXED= mixed methods; CS= cross-sectional ^3^ = “Housing” column: Type/Program: PSH= permanent supportive housing; HF = Housing First; TF = Treatment First; TAU = Treatment as Usual; SL = Sober Living Site approach: SS = single site; Scat = scattered site; Priv Mkt = private market Dwelling type: SRO = single-room occupancy; Cong = congregate housing; Apt = apartment; Ind = independent; Shrd = shared ^4^ = “Spatial Scale” column: D = dwelling unit; R = room (shared common area); B = building; L = location						

## Supplementary Materials

The following are available online at https://www.mdpi.com/article/10.3390/ijerph18189588/s1, Table S1: Databases and search terms: Complete search syntax. Table S2a: Summary of built environment (BE) findings at the room scale (dwelling unit and shared space); Table S2b: Summary of built environment (BE) findings at the building and location scales; Table S3: Built environment attributes addressed by the reviewed literature; Table S4: Place attributes addressed by the reviewed literature.

## Appendix A

**Table A1:** Categories and definitions of data extracted from the reviewed literature.

Extracted Data	Categories and Definitions
Housing type (Table 3)	Permanent supportive housing (PSH) provides affordable, safe, and stable housing to people experiencing homelessness, mental and substance use disorders, and/or disability (see Background).
Service model (Table 3)	Housing First (HF) offers immediate housing and supportive services to individuals regardless of substance use and psychiatric treatment status; individuals are encouraged to define their own recovery-oriented goals [1,24]. Treatment First (TF), also referred to as Sober Living, Sober Living Housing, and Treatment as Usual, offers temporary housing to individuals experiencing homelessness, mental illness, and/or substance use issues with the requirement that they receive treatment and progress through a hierarchy of housing options based on “housing readiness” [1].
Site approach [1] (Table 3)	Single site (SS) refers to one dedicated site (through new purpose-built construction, purchase of an existing building, or a master lease of an existing building) that primarily serves formerly homeless individuals with service needs. Dwelling units can be located within the same building, block, or neighborhood, and supportive services are usually available on site. Dwelling units can be independent apartments, shared independent apartments, shared rooms, or SROs with shared bathrooms kitchens. Scattered site (Scat) refers to private market apartments or general affordable housing dispersed throughout the community and leased by residents who are no longer experiencing homelessness via rental subsidies. Supportive staff may visit dwellings or provide services off-site.
Dwelling unit type (Table 3 and Table S1a,b)	Independent apartments: A dwelling unit with a lockable entry door, full bathroom, kitchen with refrigeration and cooking capability, and living/sleeping areas (e.g., a studio/one-bedroom apartment). Shared independent apartment: An independent apartment that is shared by two or more individuals. Congregate housing: Clustered arrangements of individual sleeping rooms with shared bathroom and kitchen facilities for each cluster of several residents. Rooms can be shared or single-occupancy. SRO (single-room occupancy): A single-occupancy dwelling unit with a bed that may or may not have a sink or small refrigerator. An SRO usually does not have its own bathroom or cooking facilities; instead, common bathrooms and cooking areas are shared.
Spatial scales (Table 3 and Table S4)	Room scale: Interior spaces that were divided into two subscales. Dwelling units referred to individual or shared rooms and apartments where residents lived. Shared common spaces referred to areas such as community rooms, kitchens, media rooms, laundry facilities, and outdoor spaces. Building scale: The building scale included factors such as the overall floor plan layout, adjacencies and arrangements of spaces, and the number of units. Location scale: Factors related to PSH location including access to transportation, amenities, and services; condition; and safety.
Built environment properties [68] (Table S2)	Ambient properties: Environmental conditions relating to the senses, also often included in measures of indoor environmental quality, such as lighting, sound, odor, temperature, humidity and ventilation. Physical properties: Built or natural elements that create and are contained within space such as the structure and enclosure (floors, walls, roof, windows), environmental control systems (heating and cooling, plumbing, electric, security), furnishings, fixtures, equipment, materials, and finishes. This paper included single and aggregated measures of quality (housing, environmental, and physical) and condition in this category. Spatial properties: Quantifiable spatial characteristics within and between physical spaces such as size, shape, proportion, volume, spatial and social densities, adjacency, proximity, layout, and arrangement.

**Table A2:** Place attribute definitions and references.

Place Attribute	Definition	Reference
Safety and security	A state in which hazards and environmental conditions leading to physical, psychological, and material harm are controlled in order to preserve individual and group well-being. Security is the process and equipment that protects safety and well-being. Alternate definitions of security referring to maintaining consistent possession of personal items and housing were not coded as place attributes.	[109]
Control	The extent to which an environment facilitates personalization and territorial claims to a space, as well as the ability to alter one’s physical environment or regulate exposure to surroundings.	[68,69]
Choice	The provision of options in the physical environment, such as the ability to select a space or pathway (spatial choice), that leads to positive outcomes.	[110]
Privacy	The process of regulating the flow of visual and auditory information to and from others. When regulatory processes (e.g., territoriality, personal space) fail, social isolation and withdrawal can occur. Social isolation is the absence of positive social relationships resulting from restriction of contact with most or all other people (and/or activity, services, programs, stimulation) and is imposed by others. Social withdrawal is the restriction of contact with most or all other people (and/or activity, services, programs, stimulation) and is self-imposed due to, for example, over-stimulation or fear of harm (e.g., violence, substance abuse influences).	[68,94,95,111]
Territoriality	A boundary-regulation mechanism used to achieve desired levels of privacy that involves personalization or marking of a place or object and communicating that the place or object is “owned” by a person or group.	[94]
Sociality	The degree to which an environment facilitates or inhibits social interaction among people.	[68]
Sense of community	The feelings of belonging or affiliation to a group, that individual members matter to each other and the group, and that members’ needs will be met via commitment to that group.	[112,113]
Sense of “home”	A place for refuge, protection, security, safety, and centering described by comfort, privacy, familiarity, multiple layers of meaning, and a sense of self-expression, identity, responsibility, ownership, and being “at one” within the setting; the absence of mistreatment, alienation, and discomfort.	[70,85]
Comfort	The extent to which an environment provides both sensory and mobility fit and facilitates task performance.	[68]
Legibility	The ease with which people can conceptualize key spatial relationships within an environment.	[68]
Wayfinding	How people orient themselves and navigate to destinations in spaces within rooms, buildings, and cities.	[68]
Accessibility	The ease in locomotion through and use of an environment or space by users of varying abilities; addressed by ADA, universal, inclusive, and barrier-free design.	[68]
Adaptability	The ease with which an environment or space and its components can be reorganized to accommodate different patterns of use.	[68]
Sensory stimulation	The quality and quantity of information in a setting or object that impinges upon human users as experienced by the various senses.	[68,69]
Restoration	An environment’s ability to provide relief and recovery from mental fatigue often resulting from overstimulation and stress.	[102]
Crowding	The psychological response to high density based on perceptions of spatial restriction due to too little space (spatial density) or too many people present in a space (social density).	[114]
Meaning	The extent to which an environment holds individual or collective significance for people (e.g., attachment, challenge, beauty).	[68]

## Appendix B

**Table A3:** Overview of housing quality measures and associated outcomes from the reviewed literature.

Citation	Housing Quality Measure (Data Collection Methods)	Subscales (# items)	Measure Reference	Outcomes Related to Mental Health
Adair et al., 2016 [38]	Observer-Rated Housing Quality Scale (Observations, interview questions, publicly available data) Perceived Housing Quality Scale (Participant-rated Likert scales)	Dwelling unit (18) Building (7) Neighborhood (9) Comfort, Safety, Privacy, Spaciousness, Overall Quality, Proximity (24)	Adair, 2014 [115] Tsemberis et al., 2003 [116] Toro et al., 1997 [117]	Housing stability
Brown et al., 2015 [72]	Housing Environment Survey (Participant-rated Likert scales and 5 semi-open-ended questions)	Physical quality (11) Neigh. quality (9) Neigh. social climate (10) Neigh. safety (8) Neighbor relationships (7) Landlord relationship (6) Roommate relationship (6) *	Kloos and Shah, 2009 [118]	Housing satisfaction
Nelson et al., 2007 [74]	Housing Quality Assessment ** (Participants rated 4-point scales for their previous residence and residences at baseline and follow-up)	Comfort, Safety, Privacy, Spaciousness, Overall Quality (5)	Toro, 1997 [117]	Self-reported quality of life, adaptation to community living
Tsai 2010 [75]	Housing Environment Survey-Physical Quality Scale (Participant-rated Likert scale)	Physical Quality (14)	Wright and Kloos, 2007 [119]	Housing satisfaction, residential satisfaction, social support
	SAMSHA Housing Satisfaction Scale (Participant-rated Likert scales)	Choice, Safety, Privacy, Proximity (19)	Tsemberis et al., 2003 [116]	
Tsai 2012 [76]	SAMSHA Housing Satisfaction Scale (Participant-rated Likert scales)	Choice, Safety, Privacy, Proximity (19)	Tsemberis et al., 2003 [116]	Housing satisfaction, self-reported quality of life, mental health, social support, psychological distress

* The Housing Environment Survey Roommate Relationship subscale was omitted from the study. ** Housing quality was a dependent variable in this study and an independent variable in the other 4 articles in this table. SAMSHA = The Substance Abuse and Mental Health Services Administration.

## References

[1] National Academies of Sciences, Engineering, and Medicine (2018). Permanent Supportive Housing: Evaluating the Evidence for Improving Health Outcomes among People Experiencing Chronic Homelessness.

[2] Woodhall-Melnik J.R., Dunn J.R. (2016). A systematic review of outcomes associated with participation in Housing First programs. Hous. Stud..

[3] Henry M., de Sousa T., Roddey C., Gayen S., Bednar T.J., Associates A. (2020). The 2020 Annual Homeless Assessment Report (AHAR) to Congress, Part 1: Point-in-Time Estimates of Homelessness.

[4] Gaetz S., Dej E., Richter T., Redman M. (2016). The State of Homelessness in Canada 2016 COH Research Paper #12.

[5] Bebout R.R. (2001). Trauma-informed approaches to housing. New Dir. Ment. Health Serv..

[6] Evans G.W., Wells N.M., Chan H.-Y.E., Saltzman H. (2000). Housing quality and mental health. J. Consul Clin. Psychol..

[7] Fazel S., Khosla V., Doll H., Geddes J. (2008). The Prevalence of Mental Disorders among the Homeless in Western Countries: Systematic Review and Meta-Regression Analysis. PLoS Med..

[8] Aljunaidy M.M., Adi M.N. (2021). Architecture and Mental Disorders: A Systematic Study of Peer-Reviewed Literature. HERD Health Environ. Res. Des. J..

[9] Padgett D.K., Stanhope V., Henwood B., Stefancic A. (2010). Substance Use Outcomes Among Homeless Clients with Serious Mental Illness: Comparing Housing First with Treatment First Programs. Community Ment. Health J..

[10] Caton C.L.M., Dominguez B., Schanzer B., Hasin D.S., Shrout P.E., Felix A., McQuistion H., Opler L.A., Hsu E. (2005). Risk Factors for Long-Term Homelessness: Findings From a Longitudinal Study of First-Time Homeless Single Adults. Am. J. Public Health.

[11] Kertesz S.G., Larson M.J., Horton N.J., Winter M., Saitz R., Samet J.H. (2005). Homeless chronicity and health-related quality of life trajectories among adults with addictions. Med. Care.

[12] Padgett D.K., Gulcur L., Tsemberis S. (2006). Housing First Services for People Who Are Homeless with Co-Occurring Serious Mental Illness and Substance Abuse. Res. Soc. Work. Pract..

[13] Henry M., Watt R., Rosenthal L., Shivji A., Associates A. (2016). The 2016 Annual Homeless Assessment Report (AHAR) to Congress November 2016 PART 1: Point-in-Time Estimates of Homelessness.

[14] Bloomfield M.A. (2013). My Eyes Feel They Need to Cry: Stories from the Formerly Homeless.

[15] Wright J.D. (2009). Address Unknown: The Homeless in America.

[16] Ly A., Latimer E. (2015). Housing First impact on costs and associated cost offsets: A review of the literature. Canadian J. Psychiatry.

[17] Nelson G., MacLeod T., Sylvestre J., Nelson G., Aubry T. (2017). The evolution of housing for people with serious mental illness. Housing, Citizenship, and Communities for People with Serious Mental Illness: Theory, Research, Practice, and Policy Perspectives.

[18] Parsell C., Petersen M., Moutou O. (2015). Single-site Supportive Housing: Tenant Perspectives. Hous. Stud..

[19] Rog D.J., Marshall T., Dougherty R.H., George P., Daniels A.S., Ghose S.S., Delphin-Rittmon M.E. (2014). Permanent Supportive Housing: Assessing the Evidence. Psychiatr. Serv..

[20] Gubits D., Shinn M., Wood M., Brown S.R., Dastrup S.R., Bell S.H. (2018). What Interventions Work Best for Families Who Experience Homelessness? Impact Estimates from the Family Options Study. J. Policy Anal. Manag..

[21] Tsemberis S., Eisenberg R.F. (2000). Pathways to Housing: Supported Housing for Street-Dwelling Homeless Individuals with Psychiatric Disabilities. Psychiatr. Serv..

[22] Parsell C., Moutou O., Lucio E., Parkinson S. (2015). Supportive housing to address homelessness. AHURI Final Report No. 240.

[23] Tsemberis S. (2012). Housing First: Basic tenets of the definition across cultures. Eur. J. Homelessness.

[24] U.S. Interagency Commission on Homelessness (2017). Investing in the End of Homelessness: The President’s 2017 Budget, Fact Sheet.

[25] Ridgway P., Zipple A.M. (1990). The paradigm shift in residential services: From the linear continuum to supported housing approaches. Psychosoc. Rehabil. J..

[26] Huffman T. (2017). Built community: Architecture, community, and participation in a permanent supportive housing project. J. Soc. Distress Homeless.

[27] McLane Y., Pable J. (2020). Architectural Design Characteristics, Uses, and Perceptions of Community Spaces in Permanent Supportive Housing. J. Inter. Des..

[28] Connellan K., Gaardboe M., Riggs D., Due C., Reinschmidt A., Mustillo L. (2013). Stressed Spaces: Mental Health and Architecture. HERD: Health Environ. Res. Des. J..

[29] Sullivan C.W., Chang C.Y., Dannenberg A., Frumkin H., Jackson R. (2011). Mental health and the built environment. Making Healthy Places.

[30] Friesinger J., Topor A., Bøe T.D., Larsen I.B. (2019). Studies regarding supported housing and the built environment for people with mental health problems: A mixed-methods literature review. Health Place.

[31] Parr H. (2000). Interpreting the ‘hidden social geographies’ of mental health: Ethnographies of inclusion and exclusion in semi-institutional places. Health Place.

[32] Halpern D. (1995). Mental Health & the Built Environment: More Than Bricks & Mortar.

[33] Frumkin H. (2003). Healthy places: Exploring the evidence. Am. J. Public Health.

[34] Evans G.W., Wells N.M., Moch A. (2003). Housing and Mental Health: A Review of the Evidence and a Methodological and Conceptual Critique. J. Soc. Issues.

[35] Johansson M., Brunt D. (2012). The Physical Environment of Purpose-Built and Non-Purpose-Built Supported Housing for Persons with Psychiatric Disabilities in Sweden. Issues Ment. Health Nurs..

[36] Knight K.R., Lopez A.M., Comfort M., Shumway M., Cohen J., Riley E.D. (2014). Single room occupancy (SRO) hotels as mental health risk environments among impoverished women: The intersection of policy, drug use, trauma, and urban space. Int. J. Drug Policy.

[37] Pevalin D.J., Reeves A., Baker E., Bentley R. (2017). The impact of persistent poor housing conditions on mental health: A longitudinal population-based study. Prev. Med..

[38] Adair C.E., Kopp B., Distasio J., Hwang S.W., Lavoie J., Veldhuizen S., Voronka J., Kaufman A.F., Somers J.M., Leblanc S.R. (2016). Housing Quality in a Randomized Controlled Trial of Housing First for Homeless Individuals with Mental Illness: Correlates and Associations with Outcomes. J. Hered..

[39] Liddell C., Guiney C. (2015). Living in a cold and damp home: Frameworks for understanding impacts on mental well-being. Public Health.

[40] Rautio N., Filatova S., Lehtiniemi H., Miettunen J. (2018). Living environment and its relationship to depressive mood: A systematic review. Int. J. Soc. Psychiatry.

[41] Shah S.N., Fossa A., Steiner A., Kane J., Levy J.I., Adamkiewicz G., Bennett-Fripp W.M., Reid M. (2018). Housing Quality and Mental Health: The Association between Pest Infestation and Depressive Symptoms among Public Housing Residents. J. Hered..

[42] Lindberg R.A., Shenassa E.D., Acevedo-Garcia D., Popkin S.J., Villaveces A., Morley R.L. (2010). Housing Interventions at the Neighborhood Level and Health. J. Public Health Manag. Pract..

[43] Sommer R. (1967). Sociofugal Space. Am. J. Sociol..

[44] Marcus C.C. (1995). House as a Mirror of Self: Exploring the Deeper Meaning of Home.

[45] Baum A., Valins S. (1977). Architecture and Social Behavior: Psychological Study of Social Density.

[46] Baum A., Valins S. (1979). Architectural mediation of residential density and control: Crowding and the regulation of social contact. Adv. Exp. Soc. Psychol..

[47] Baum A., Gatchel R. (1981). Cognitive mediation of environmental stress. Cognition, Social Behavior, and the Environment.

[48] Hillier B., Hanson J. (1984). The Social Logic. of Space.

[49] Festinger L., Schachter S., Back K. (1950). Social Pressures in Informal Groups; a Study of Human Factors in Housing.

[50] Wells M.N., Rollings K.A., Clayton S. (2012). The natural environment in residential settings: Influences on human health and function. The Oxford Handbook of Environmental and Conservation Psychology.

[51] Pable J. (2007). Interior Design Homeless shelter design: A psychologically recuperative approach. J. Inter. Des..

[52] Pable J. (2012). The Homeless Shelter Family Experience: Examining the Influence of Physical Living Conditions on Perceptions of Internal Control, Crowding, Privacy, and Related Issues. J. Inter. Des..

[53] Pable J. (2013). Possessions in the homeless shelter experience: The built environment’s potential role in self-restoration. Interiors.

[54] Liddicoat S., Badcock P., Killackey E. (2020). Principles for designing the built environment of mental health services. Lancet Psychiatry.

[55] Shepley M.M., Pasha S. (2017). Design for Mental and Behavioral Health.

[56] Shepley M.M., Pasha S. (2013). Design Research and Behavioral Health Facilities.

[57] Lundqvist L.-O., Rask M., Brunt D., Ivarsson A.-B., Schröder A. (2016). Measuring quality in community based housing support—The QPC-H instrument. Int. J. Health Care Qual. Assur..

[58] Marcheschi E., Laike T., Brunt D., Hansson L., Johansson M. (2015). Quality of life and place attachment among people with severe mental illness. J. Environ. Psychol..

[59] Marcheschi E., Johansson M., Laike T., Brunt D. (2016). Housing design and people with severe mental illness: An observational approach to the investigation of supported housing facilities. Scand. J. Psychol..

[60] Whittemore R., Knafl K. (2005). The integrative review: Updated methodology. J. Adv. Nurs..

[61] Miles B.M., Huberman A.M. (1994). Qualitative Data Analysis.

[62] Bollo C., Donofrio A. From principles to patterns: Trauma-informed design for Permanent Supportive Housing. Housing and Society.

[63] Goering P.N., Streiner D., Adair C., Aubry T., Barker J., Distasio J., Hwang S.W., Komaroff J., Latimer E., Somers J. (2011). The At Home/Chez Soi trial protocol: A pragmatic, multi-site, randomised controlled trial of a Housing First intervention for homeless individuals with mental illness in five Canadian cities. BMJ Open.

[64] Clark C., Myron R., Stansfeld S., Candy B. (2007). A systematic review of the evidence on the effect of the built and physical environment on mental health. J. Public Ment. Health.

[65] Greenwood R.M., Manning R.M., O’Shaughnessy B.R., Vargas-Moniz M.J., Loubière S., Spinnewijn F., Lenzi M., Wolf J.R., Bokszczanin A., Bernad R. (2020). Homeless Adults’ Recovery Experiences in Housing First and Traditional Services Programs in Seven European Countries. Am. J. Community Psychol..

[66] Page M.J., McKenzie J.E., Bossuyt P.M., Boutron I., Hoffmann T.C., Mulrow C.D., Shamseer L., Tetzlaff J.M., Akl E.A., Brennan S.E. (2021). The PRISMA 2020 statement: An updated guideline for reporting systematic reviews. BMJ.

[67] Garrard J. (2016). Health Sciences Literature Review Made Easy: The Matrix Method.

[68] Weisman G. (2001). The place of people in architectural design. Architectural Design Portable Handbook: A Guide to Excellent Practices.

[69] Evans G.W., McCoy J.M. (1998). When buildings don’t work: The role of architecture in human health. J. Environ. Psychol..

[70] Rivlin L.G., Moore J. (2001). Home-Making: Supports and Barriers to the Process of Home. J. Soc. Distress Homeless.

[71] Wittman F.D., Polcin U.L., Sheridan D. (2017). The architecture of recovery: Two kinds of housing assistance for chronic homeless persons with substance use disorders. Drugs Alcohol Today.

[72] Brown M., Malone D., Jordan N. (2015). Tenant Satisfaction with a Single-Site Housing First Program. J. Soc. Serv. Res..

[73] Hsu H.-T., Simon J.D., Henwood B.F., Wenzel S.L., Couture S.L.W. (2016). Location, Location, Location: Perceptions of Safety and Security Among Formerly Homeless Persons Transitioned to Permanent Supportive Housing. J. Soc. Soc. Work. Res..

[74] Nelson G., Sylvestre J., Aubry T., George L., Trainor J. (2006). Housing Choice and Control, Housing Quality, and Control over Professional Support as Contributors to the Subjective Quality of Life and Community Adaptation of People with Severe Mental Illness. Adm. Policy Ment. Health Ment. Health Serv. Res..

[75] Tsai J., Bond G.R., Davis K.E. (2010). Housing Preferences Among Adults with Dual Diagnoses in Different Stages of Treatment and Housing Types. Am. J. Psychiatr. Rehabil..

[76] Tsai J., Mares A.S., Rosenheck R.A. (2011). Housing Satisfaction Among Chronically Homeless Adults: Identification of its Major Domains, Changes Over Time, and Relation to Subjective Well-being and Functional Outcomes. Community Ment. Health J..

[77] Anucha U. (2005). We are not just rent receipts: Housing, neighbourhood, and community re-imagined by formerly homeless people. Rev. Can. Serv. Soc..

[78] Burns V.F., LeDuc J.D.-, St-Denis N., Walsh C.A. (2019). Finding home after homelessness: Older men’s experiences in single-site permanent supportive housing. Hous. Stud..

[79] Chan D.V. (2018). Safe Spaces, Agency, and Connections to “Regular Stuff”: What Makes Permanent Supportive Housing Feel Like “Home”. Rehabil. Couns. Bull..

[80] Henwood B.F., Lahey J., Harris T., Rhoades H., Wenzel S.L. (2018). Understanding Risk Environments in Permanent Supportive Housing for Formerly Homeless Adults. Qual. Health Res..

[81] Henwood B.F., Redline B., Semborski S., Rhoades H., Rice E., Wenzel S.L. (2018). What’s next? A theory on identity preservation for young adults in supportive housing. Cityscape.

[82] Padgett D.K. (2007). There’s no place like (a) home: Ontological security among persons with serious mental illness in the United States. Soc. Sci. Med..

[83] Adame A.L., Perry C., Pierce E. (2020). Community and Housing First: A qualitative analysis of USA residents’ perspectives. Health Soc. Care Comm..

[84] Rykwert J. (1991). House and home. Soc. Res..

[85] Rowles G.D., Devlin A.S. (2018). Housing for older adults. Environmental Psychology and Human Well-Being: Effects of Built and Natural Settings.

[86] Dupuis A., Thorns D.C. (1998). Home, Home Ownership and the Search for Ontological Security. Sociol. Rev..

[87] Wong Y.I., Hadley T.R., Culhane D.P., Poulin S.R., Davis M.R. (2006). Predicting Staying in or Leaving Permanent Supportive Housing That Serves Homeless People with Serious Mental Illness.

[88] Hetling A., Dunford A., Botein H. (2020). Community in the Permanent Supportive Housing Model: Applications to Survivors of Intimate Partner Violence. Housing, Theory Soc..

[89] Bridgman R. (2002). Housing chronically homeless women: “Inside” a safe haven. Hous. Policy Debate.

[90] Hopper E.K., Bassuk E.L., Olivet J. (2010). Shelter from the Storm: Trauma-Informed Care in Homelessness Services Settings. Open Health Serv. Policy J..

[91] Fallot D.R., Harris M. (2008). Trauma-informed approaches to systems of care. Trauma Psychol. Newsl..

[92] Farrell J. (2018). The Committee on Temporary Shelter. Trauma-Informed Design: How the Physical Environment Supports Recovery from Homelessness. http://cotsonline.org/wp-content/uploads/2018/04/Trauma-Informed-Design.BOD_.pdf.

[93] Henwood B.F., Lahey J., Rhoades H., Winetrobe H., Wenzel S.L. (2017). Examining the health status of homeless adults entering permanent supportive housing. J. Public Health.

[94] Altman I. (1975). The Environment and Social Behavior: Privacy, Personal Space, Territory, Crowding.

[95] Cacioppo T.J., Cacioppo S. (2014). Social relationships and health: The toxic effects of perceived social isolation. Soc. Personal. Psychol. Compass.

[96] Appleton J. (1975). The Experience of Landscape.

[97] Evans G.W., Rhee E., Forbes C., Allen K.M., Lepore S. (2000). The meaning and efficacy of social withdrawal as a strategy for coping with chronic residential crowding. J. Environ. Psychol..

[98] Li D., Menotti T., Ding Y., Wells N. (2021). Life Course Nature Exposure and Mental Health Outcomes: A Systematic Review and Future Directions. Int. J. Environ. Res. Public Health.

[99] Devlin A.S. (2014). Wayfinding in Healthcare Facilities: Contributions from Environmental Psychology. Behav. Sci..

[100] The Center for Universal Design (1997). The Principles of Universal Design.

[101] Henwood B.F., Lahey J., Rhoades H., Pitts D.B., Pynoos J., Brown R.T. (2019). Geriatric Conditions Among Formerly Homeless Older Adults Living in Permanent Supportive Housing. J. Gen. Intern. Med..

[102] Kaplan S., Kaplan R. (1989). Cognition and Environment: Functioning in an Uncertain World.

[103] Schweitzer M., Gilpin L., Frampton S. (2004). Healing Spaces: Elements of Environmental Design That Make an Impact on Health. J. Altern. Complement. Med..

[104] Abdelaal M.S., Soebarto V. (2019). Biophilia and Salutogenesis as restorative design approaches in healthcare architecture. Arch. Sci. Rev..

[105] Aboujaoude E., Salame W., Naim L. (2015). Telemental health: A status update. World Psychiatry.

[106] Tsai J., Rosenheck R.A. (2011). Racial Differences Among Supported Housing Clients in Outcomes and Therapeutic Relationships. Psychiatr. Q..

[107] Omerov P., Craftman Å.G., Mattsson E., Klarare A. (2020). Homeless persons’ experiences of health- and social care: A systematic integrative review. Health Soc. Care Community.

[108] Henwood B.F., Cabassa L.J., Craig C.M., Padgett D.K. (2013). Permanent Supportive Housing: Addressing Homelessness and Health Disparities?. Am. J. Public Health.

[109] Maurice P., Lavoie M., Laflamme L., Svanström L., Romer C., Anderson R. (2001). Safety and safety promotion: Definitions for operational developments. Inj. Control. Saf. Promot..

[110] Gifford R. (1987). Environmental Psychology: Principles and Practice.

[111] Wener R.E., Devlin A.S. (2018). Can correctional environments be humane? A case for evidence and value-based design. Environmental Psychology and Human Well-Being: Effects of Built and Natural Settings.

[112] McMillan W.D., Chavis D.M. (1986). Sense of community: A definition and theory. J. Community Psychol..

[113] Francis J., Giles-Corti B., Wood L., Knuiman M. (2012). Creating sense of community: The role of public space. J. Environ. Psychol..

[114] Rollings K.A., Evans G.W. (2019). Design Moderators of Perceived Residential Crowding and Chronic Physiological Stress Among Children. Environ. Behav..

[115] Adair C.E., Kopp B., Lavoie J., Distasio J., Hwang S.W., Watson A., Veldhuizen S., Chislett K., Voronka J., Ahmad M. (2014). Development and Initial Validation of the Observer-Rated Housing Quality Scale (OHQS) in a Multisite Trial of Housing First. J. Hered..

[116] Tsemberis S., Rogers E.S., Rodis E., Dushuttle P., Skryha V. (2003). Housing satisfaction for persons with psychiatric disabilities. J. Community Psychol..

[117] Toro P.A., Rabideau J.M.P., Bellavia C.W., Daeschler C.V., Wall D.D., Thomas D.M., Smith S.J. (1997). Evaluating an intervention for homeless persons: Results of a field experiment. J. Consul Clin. Psychol..

[118] Kloos B., Shah S. (2009). A Social Ecological Approach to Investigating Relationships Between Housing and Adaptive Functioning for Persons with Serious Mental Illness. Am. J. Community Psychol..

[119] Wright P.A., Kloos B. (2007). Housing environment and mental health outcomes: A levels of analysis perspective. J. Environ. Psychol..

