# Supplementary material for: Permanent Supportive Housing Design Characteristics Associated with the Mental Health of Formerly Homeless Adults in the U.S. and Canada: An Integrative Review
Source: Int J Environ Res Public Health. 2021 Sep 12;18(18):9588. doi: 10.3390/ijerph18189588 (PMC8465794; doi:10.3390/ijerph18189588)
Supplement: Supplementary file 1 [file ijerph-18-09588-s001.zip › ijerph-1338962-supplementary.pdf]

Table S1. Databases and search terms: Complete search syntax.

Search Concepts	Search Terms [1]
Supportive(ed) Housing [2]	Housing, residential
Mental Health	"Mental health" OR "mental illness" OR "mental illnesses" OR "mental disorder" OR "mental disorders" OR "mental wellbeing" OR "mental well-being" or "mental well being" OR "psychological illness" OR "psychological illnesses" OR "Behavioral health" OR "psychiatric disabilities" OR Loneliness OR Trauma OR "Psychological health"
Architecture/Design	"Built Environment" OR "Interior Design" OR Architecture OR "Physical environment" OR "Environmental design" OR "environment design" OR "Design attributes" OR "design features" OR Architectural OR "Spatial characteristics" OR "design characteristics" OR Safety OR Security OR Surveillance OR Wayfinding OR Territoriality OR Crowding OR Privacy OR "housing quality" OR "environment quality" OR "environmental quality"
Excluded	(Children OR "older adults" OR "later life" OR elderly OR aged OR disabled OR "older people" OR "care facilities" OR "board home" OR "care home" OR "nursing home" OR "nursing homes" OR city OR cities OR urban OR "eating disorder" OR "eating disorders" OR "mental retardation" OR prison OR prisons OR jail OR jails OR HIV OR AIDS OR refugee OR refugees OR asylum OR youth OR adolescent OR adolescents OR workplace OR workplaces OR Covid-19)

[1] Unless noted below, search terms were applied to each of 9 databases: CINAHL Health Source, JSTOR, ProQuest, PsycInfo, PubMed, Scopus, Social Service Abstracts, and Web of Science.

- PubMed: The keyword string, (“supportive housing” or “supported housing”) was used in order to identify relevant and narrow results to a reasonable number for review.
- JSTOR limits search keywords to 200 characters, therefore two searches were run: (1) The first keyword search (“supportive housing” or “supported housing”) was limited to Architecture & Architectural History articles and (2) the second keyword search (("supported housing") OR ("supportive housing")) AND (("physical environment") OR ("built environment") OR (architecture)) was limited to Sociology, Social Work, Public Health, Psychology, and Health Science articles. Both searches were limited to articles published in English.

[2] Search terms relating to homelessness were not applied to avoid narrowing search results.

Table S2a. Summary of built environment (BE) findings at the room scale (dwelling unit and shared space).

Built Environment Findings: Room Scale

Citation (Relevance-Rigor Rating) | BE Findings

Dwelling Unit Scale Findings

Wittman et al., 2017 [71] (DD-Low)
- SLH **room size** should fit more than one person for monitoring/preventing relapse.
- HF facilities with **indep. apartments** should **promote interaction** in shared spaces.

Adair et al., 2016 [38] (BE-High)
- **Unit type and size** were stronger correlates of housing quality.
- **Unit (then neighborhood and building) aspects** were most important to participants.
- **Rooming house rooms, SROs,** and **smaller units** were associated with lower mean housing quality scores likely due **to fewer/shared amenities and less privacy**.

Knight et al., 2014 [36] (BE-High)
- **Trauma-informed SROs with en suite bathrooms** that were newly constructed (vs. old, poorly maintained SROs with shared bathrooms) provided **more control, independence, safety, and security**, and led to better mental health outcomes.

Nelson et al., 2007 [74] (BE-Med)
- **Independent apartment residents** (vs. those residing in group living arrangements) reported significantly higher levels of **housing choice, housing control,** and control over professional support, but not higher levels of housing quality.

Tsai et al., 2010 [75] (BE-High)
- **Independent apartments or houses** were preferred by dual-diagnoses consumers across all treatment stages.
- **SRO** residents (vs. residential program and apartment residents) reported the highest rents and lowest choice, satisfaction, and physical housing quality, likely due to small spaces, short-term leases, and transient neighbors.
- **Preferred (vs. other) housing type** was associated with significantly greater resident-reported choice over – but not satisfaction with - housing and activities.

Anucha, 2005 [77] (IN-Low)
- Shared housing tenants desired **bigger rooms, more space, and more privacy** and prefer their own **independent apartment** rather than "living in one room like a jail."

Burns et al., 2020 [78] (IN-High)
- **Shared rooms and bathrooms** fostered a sense of safety, but also triggered processes of territorial exclusion (vs. agency and choice) among older male participants.
- **BE choice** can assist with addressing territorial exclusion and was associated with more reported autonomy (e.g., optional in-room refrigerators and smoking), but can also contribute to less social interaction and isolation.

Chan, 2020 [79] (IN-Med)
Feelings of "home" were associated with:
- **Safe spaces** for solitary activity-quiet/privacy/retreat/isolation.
- **Ability to have "regular stuff"** (comfortable furnishings and appliances, secure storage, own bathroom, backyard).
- **Agency to choose** and pursue goals (independence, autonomy, choice, control, territoriality, safety, lack of privacy).

Henwood et al., 2018a [80] (IN-Med)
- Having an **independent apartment** dramatically changed most participants' lives and interactions with others. The valuable distinction between public and private space affected between social and physical environment interactions.

Henwood et al., 2018b [81] (IN-Med)
- **Independent apartments/PSH** generally provided a sense of ontological security among young adults.
- **Physical and social environments** are both related closely to the concept of **ontological security** (constancy, routine, control) associated with positive mental health and wellbeing, and assisting with positive identity construction.

Padgett, 2007 [82] (IN-High)
- **Independent apartments** were associated with ontological security markers: safety, privacy, freedom (control, self-determination), ability to complete "routines of daily life" (laundry, a walk in the park); privacy and escape from noise/urban life stress, and engaging in "identity construction and repair" and considering "what's next."

Shared Space Scale Findings

McLane & Pable, 2020 [27] (DD-Med)
- **Community spaces are important** according to a majority of residents and staff, but data suggest they are underused. **Visibility**, or lack of it, is likely a key reason.
- **Community space location, wayfinding,** and **visual and physical access**, followed by **aesthetics, hominess** (views, personalization, cleanliness, acoustics, lighting, daylight,), and **size** (large enough for multiple activity zones) are the most important design factors.

Wittman et al., 2017 [71] (DD-Low)
- **Socio-petal spatial layouts for common areas** can facilitate social interaction while other spaces provide privacy for personal time, sleeping, and hygiene.

Huffman, 2018 [26] (BE-High)
- **Trauma informed design is open and "different" from institutional settings**. Intentionally designed PSH community spaces that respond to trauma affect wellbeing and provide a place for interaction, but do not address all social issues.
- **Community space was both "promising and contested."** Non-rivalrous, non-scarce resources (e.g., common areas) are not depleted via use, so they are not contested compared t, e.g., community gardens/vegetables.

Burns et al., 2020 [78] (IN-High)
- Tensions related to housing design and rules arose concerning **shared bathrooms** (accessibility and exclusion), **cafeterias** (when/what to eat and with whom), and **rec rooms** (when to interact with others).
- The **ability to cook one's own meals** and **interact with others when desired**, rather than being forced to eat prepared meals in shared spaces, was a source of autonomy and control.

Chan, 2020 [79] (IN-Med)
BE choice (activity, social interaction) is associated with common area satisfaction.

Adame et al., 2020 [83] (ME-Med)
- **The built environment provides opportunities for events and activities** that, when regularly held, help to build community, create a sense of stability, and increase familiarity and trust among residents.

Main study findings and BE conclusions informed by review analysis are illustrated; insignificant results are excluded from the table.
High/Med/Low= Methodlogical rigor rating
Relevance ratings: DD = Design-driven IN = Inductive BE = Built environment focused ME = mentions
Other abbreviations: HF = Housing First SLH= Sober living housing PSH= Permanent supportive housing

Table S2b. Summary of built environment (BE) findings at the building and location scales.

**Built Environment Findings: Building and Location Scales**<br>Rigor (High/Med/Low) | (Relevance) Citation | BE Findings	
Building Scale Findings	***Location Scale Findings***
**<u>Wittman et al., 2017</u> [71] (DD-Low)**<br>- The **setting itself is the essence of the service offered**. The setting-outcome interaction should be considered.<br>- **Architectural design should be based on resident needs and preferences**, including considering how spatial layout for interaction and privacy, room size, monitoring level, professional service availability, and neighborhood characteristics fit with housing purpose and mission.<br><br>**<u>Adair et al., 2016</u> [38] (BE-High)**<br>- **Housing quality** was positively associated with housing stability after adjusting for participant, site, housing, and community aspects.<br><br>**<u>Brown et al., 2015</u> [72] (BE-Low)**<br>- **Significantly more favorable physical quality** ratings were reported by participants who preferred to stay (vs. leave).<br>- **Safety**, **autonomy**, on-site staff, affordability, and improvement over homelessness were positive themes and **noise, infestation, lack of privacy**, crime, and drugs were negative themes related to satisfaction.<br><br>**<u>Tsai et al., 2012</u> [76] (BE-High)**<br>- **Good environment, control** and consumer choice, **physical quality,** positive case manager contact, and frequency of landlord interaction were associated with increased housing satisfaction, but **housing satisfaction decreased over time**.<br><br>**<u>Anucha, 2005</u> [77] (IN-Low)**<br>- Emergent housing themes included **physical improvements** and **better-quality housing** (aesthetics, cleanliness, painting, pest control, landscaping, air circulation and air conditioning,), better/more **security**, and reduced **stigma**.<br><br>**<u>Nelson et al., 2007</u> [74] (BE-Med)**<br>- **Housing quality** significantly declined over time, and was associated with self-reported quality of life and adaptation to community living, as was housing choice and control.<br><br>***<u>Multiple</u>***<br>-Independent apartments affect **building scale layout and design** (Adair et al., 2016 [38]; Tsai et al., 2010 [75]; Anucha, 2005 [77]; Henwood et al., 2018a [80], 2018b [77]; Padgett, 2007 [82]; Chan, 2020 [79]), as do having en suite bathrooms with SROs (Chan, 2020 [79]; Burns et al., 2020 [78], Knight et al., 2014 [36]).	**<u>Wittman et al., 2017</u> [71] (DD-Med)**<br>- **Housing location** (recreational activities, proximity to transportation, resources that support vs. hinder recovery) can facilitate and hinder program goals/purposes.<br><br>**<u>Brown et al., 2015</u> [72] (BE-Low)**<br>- **Location, nearby amenities, and public transportation** were related to satisfaction.<br>- **Significantly more favorable neighborhood quality** ratings were reported by participants who preferred to stay (vs. leave).<br><br>**<u>Hsu et al., 2016</u> [73] (BE-Low)**<br>- **Skid Row was viewed as less safe** than the immediately surrounding neighborhood.<br>- Skid Row residents perceived social and physical disorder, and the area triggered past traumatic experiences while homeless.<br>- **Perceptions corresponded with observed neighborhood condition differences in physical (and social) disorder**: more trash, malodors, and presence of homeless people were recorded in Skid Row.<br>- Many individuals housed in Skid Row described preferring to spend more time in their housing units than outdoors because of neighborhood safety concerns.<br>-Participants reported an increase in perceived safety and security after transitioning into PSH, but objective and perceived neighborhood physical and social characteristics affect those perceptions.<br><br>**<u>Padgett, 2007</u> [82] (IN-High)**<br>- Neighborhood factors such as being able to walk in a park contribute to ontological security and being able to complete daily routines.<br><br>**<u>Tsai et al., 2012</u> [76] (BE-High)**<br>- Geographic proximity to desirable resources was associated with increased housing satisfaction.<br><br>**<u>Anucha, 2005</u> [77] (IN-Low)**<br>- Emergent neighborhood themes included **stigma, safety** and **security**, **noise** and **traffic**, **aesthetics** (garbage, lack of trees/plants), and **location** (transportation, traffic, and proximity to slaughterhouse).<br>- Participants suggested various **service and facility improvements**, such as increasing facilities like parks and swimming.

Main study findings and BE conclusions informed by review analysis are illustrated; insignificant results are excluded from the table.
High/Med/Low= Methodlogical rigor rating
Relevance ratings: DD = Design-driven IN = Inductive BE = Built environment focused ME = mentions

Table S3. Built environment attributes addressed by the reviewed literature.

Citation	BE RELEVANCE	*Physical & Ambient Properties*: Condition/quality*	Upkeep	Outdoor space	Acoustics	Ambient	Storage	Aesthetics & design	Shelter & facilities	Security measures	Shared space	Furniture & equip.	Wayfinding items	*Spatial Properties*: "Own space"	Common space	Own vs. shared room	Dwelling size	Apartment vs. room	En suite vs. hall bath	Layout	Floor level	Building type	*Location Properties*: Amenity proximity	Location	Public transportation	Condition/quality	Safety/security	Noise
**McLane et al., 2020** [27]	DD		•		•	•							•		•													
**Wittman et al., 2017** [71]	DD		•				•	•							•	•				•			•	•	•			
**Adair et al., 2016** [38]	BE	•	•	•	•	•	•	•	•					•		•	•					•	•		•	•		
**Brown et al., 2015** [72]	BE	•	•		•																		•	•	•			
**Hsu et al., 2016** [73]	BE						•			•				•										•		•		
**Huffman, 2018** [26]	BE			•							•				•					•	•							
**Knight et al., 2014** [36]	BE	•	•							•									•	•	•		•		•			
**Nelson et al., 2007** [74]	BE	•															•	•										
**Tsai et al., 2010** [75]	BE	•	•											•			•	•					•					
**Tsai et al., 2012** [76]	BE	•		•																			•				•	
**Anucha, 2005** [77]	IN	•	•	•		•		•						•		•	•	•					•	•	•	•	•	•
**Burns et al., 2020** [78]	IN			•	•		•	•				•			•	•			•									
**Chan, 2020** [79]	IN			•	•		•					•		•	•				•									
**Henwood et al., 2018a** [80]	IN															•							•	•	•			
**Henwood et al., 2018b** [81]	IN								•					•														
**Padgett, 2007** [82]	IN								•					•									•					
**Adame et al., 2020** [83]	ME			•	•						•				•								•				•	
**TOTAL articles**		7	7	7	6	5	5	4	3	2	2	2	1	7	6	5	4	3	3	3	2	1	10	5	6	3	3	1

BE Relevance: DD = Design-driven; BE = Built environment focused; IN = Inductive; ME = mentions

* = Includes 5 studies with aggregated measures of housing quality.

Sixteen articles addressed physical and ambient properties (Table S2) including general physical condition and quality (7); upkeep (7-cleanliness, pests, maintenance); the presence, condition, and quality of outdoor space (7-landscaping, gardens, backyard, smoking area, access to nature); acoustics (6-noise, quiet); ambient environment (5-air circulation, air conditioning, lighting, plumbing, power, heating); storage (5-availability and ability to lock); aesthetics (4-appearance, design, paint condition); shelter and facilities (3-having a roof and shower, cooking/kitchen, and laundry facilities); security measures (2-cameras, locks, fences); shared space (2-quality, visibility via glass walls); furniture and equipment (2-furnishings, appliances, in-room refrigerator); and wayfinding items (1-signage, colors, distinguishable designs by floor). Similarly, spatial properties were addressed by a total of 15 studies (Table S1). These properties included having one's "own space" (7); common space (6-quantity, size, layout, and location, including with respect to visibility and noise); having one's own versus a shared room for privacy (5); dwelling size and spaciousness (4); having an apartment versus a room with shared bathroom and cooking facilities (3); having an "en suite" or connected bathroom versus a shared bathroom "down the hall" (3); building layout (3-open space and visibility with respect to sociability and monitoring); floor level (2); and building type (1-high rise, low rise, fourplex, duplex, house). Place attributes were addressed by all 17 studies included in the review (Table S3).

Table S4. Place attributes addressed by the reviewed literature.

Citation	BE RELEVANCE	Safety	Security	Control	*Choice*	Privacy	*Isolation*	Territoriality	Sociality	Sense of Community	Sense of 'Home'	Comfort	Independence	Ont. Security	*Identity*	Autonomy	Trauma Sensitivity
		Place Attributes (Physical + Social Properties)															
**McLane et al., 2020** [27]	DD	•		•	•	•	•		•	•	•	•			•		•
**Wittman et al., 2017** [71]	DD	•	•			•	•		•								
**Adair et al., 2016** [38]	BE	•	•		•	•										•	
**Brown et al., 2015** [72]	BE	•	•			•										•	
**Hsu et al., 2016** [73]	BE	•	•	•			•	•				•		•			•
**Huffman, 2018** [26]	BE							•	•	•	•				•		•
**Knight et al., 2014** [36]	BE	•	•	•			•					•	•			•	•
**Nelson et al., 2007** [74]	BE	•		•	•	•						•					
**Tsai et al., 2010** [75]	BE	•			•	•							•			•	
**Tsai et al., 2012** [76]	BE	•	•	•	•	•										•	
**Anucha, 2005** [77]	IN	•	•			•					•	•	•			•	
**Burns et al., 2020** [78]	IN	•	•	•	•	•		•	•	•	•		•		•	•	
**Chan, 2020** [79]	IN	•		•	•	•	•	•	•		•	•	•	•	•	•	
**Henwood et al., 2018a** [80]	IN			•		•	•		•						•		
**Henwood et al., 2018b** [81]	IN			•		•	•				•			•	•		
**Padgett, 2007** [82]	IN	•		•		•					•		•	•	•		
**Adame et al., 2020** [83]	ME	•		•			•	•	•	•	•	•					•
**TOTAL articles**		**14**	**8**	**11**	**7**	**13**	**8**	**5**	**7**	**4**	**8**	**7**	**6**	**4**	**7**	**9**	**5**

BE Relevance: DD = Design-driven IN = Inductive
BE = Built environment focused ME = mentions
Other abbreviations: TID = Trauma informed design Ont. Security = Ontological security
